# Diesel particulate matter-induced proteomic changes in three-dimensional spheroids derived from human primary cells of various tissue origins

**DOI:** 10.1038/s41598-025-17873-7

**Published:** 2025-09-24

**Authors:** Yoon Jin Cho, Hae Dong Jeong, Young June Jeon, Soobin Choi, Ji Hyun Back, Ji Hun Wi, Yae Eun Park, Seung-Hee Gwak, Mi Jung Ji, Hyun-Mee Park, Hyuk Jeong, So Yeon Kim, Ji Eun Lee

**Affiliations:** 1 Chemical and Biological Integrative Research Center, Biomedical Research Division, Korea Institute of Science and Technology, Seoul, 02792 Republic of Korea; 2 Department of Chemistry, Sookmyung Women’s University, Seoul, 04310 Republic of Korea; 3 Department of Bioengineering, Hanyang University, Seoul, 04763 Republic of Korea; 4 Department of Biotechnology, College of Life Sciences and Biotechnology, Korea University, Seoul, 02841 Republic of Korea; 5 Advanced Analysis and Data Center, Research Resources Division, Korea Institute of Science and Technology, Seoul, 02792 Republic of Korea; 6 Division of Bio-Medical Science and Technology, KIST School, University of Science and Technology (UST), Seoul, 02792 Republic of Korea; 7 KHU-KIST Department of Converging Science and Technology, Kyung Hee University, Seoul, 02447 Republic of Korea

**Keywords:** Three-dimensional (3D) spheroids, Viability assessment, Diesel particulate matter (DPM), Tandem mass tag (TMT), Mass spectrometry (MS), Biomarkers, Cell signalling

## Abstract

This study investigated the effects of diesel particulate matter (DPM) on multiple human organs using 3D spheroids derived from eight human primary cell types. To assess the impact of DPM, we exposed these spheroids to varying concentrations of standardized DPM (Standard Reference Material, SRM 2975) and measured their viability, followed by proteomic analysis using tandem mass tag (TMT) labeling with liquid chromatography–tandem mass spectrometry (LC-MS/MS). A total of 9,707 proteins were identified, with 128 proteins exhibiting statistically significant changes (*P*-value < 0.05) in response to DPM exposure, as determined by two-way analysis of variance (ANOVA). Among these, five proteins, including apolipoprotein A-I (APOA1), significantly increased at higher DPM concentrations, while 36 proteins, primarily ribosomal proteins, showed notable decreases even at lower DPM levels. Canonical pathway analysis revealed activation of acute phase response signaling, liver X receptor/retinoid X receptor (LXR/RXR), and farnesoid X receptor (FXR)/RXR pathways across all spheroid types. APOA1 was identified as a potential biomarker for DPM exposure, with increased expression potentially linked to disruption in protein degradation pathways. This study provides valuable insights into the systemic toxicity of DPM, highlighting key proteomic changes across different tissue types and identifying potential biomarkers that could be used to assess exposure levels and health risks related to particulate matter.

**Supplementary Information:**

The online version contains supplementary material available at 10.1038/s41598-025-17873-7.

## Introduction

Air pollution has posed a significant global public health concern. Numerous studies have investigated the effects of air pollutants primarily on the respiratory system, due to the fact that air pollutants enter the human body through breathing. However, it is well reported that particulate matter (PM), especially smaller than 2.5 μm in size, can translocate to bloodstream, be deposited in various organs including liver, spleen, and brain, and therefore increase the risk of many diseases, ultimately affecting human life quality and lifespan^1,2^.

To elucidate the effects of PM on human health and diseases, several approaches have been utilized. Epidemiological studies can be useful to elucidate correlations between the exposure of air pollution and various diseases such as cardiovascular disease, autoimmune disorders, and malfunctions of lung and circulatory systems^2^. However, it should be noted that the associations between PM exposure and health outcomes in observational epidemiological studies can be affected by other factors, including individual behaviors (such as smoking and drinking), socioeconomic status, and neighborhood characteristics. Animal models, usually rodent models, help reveal the detailed mechanisms underlying disease progression, and the relationship between exposure dose and the effects of PM often corroborates with the epidemiologic studies^3^. Still, ethical issues and pathophysiological differences restrict the use of animal models^4^. Due to its ease and rapidity, in vitro two-dimensional (2D) cultures with human cells have been utilized to elucidate the signaling pathways and underlying mechanisms of PM exposure, and the integration of in vitro and in vivo responses to PM has been explored^5^. However, cells within intact tissue are enclosed in an extracellular matrix, blood vessels, and other cells, and therefore their response to PM in vivo may differ from what is observed in 2D cultures. For example, the exposure and subsequent penetration of PM in vivo is proportionally increased in the exposed outer regions of the three-dimensional (3D) surface. For these reasons, various 3D models, including air-liquid interface cultures, organ-typical cell cultures, organoids, and organ-on-a-chip systems, have been developed to study the impact of PM on airway diseases^6^.

As stated above, studies have investigated the effects of PM on respiratory diseases using 3D models. However, given the fact that PM can affect all organs of the human body, there are few studies that have examined the comprehensive effects of PM on multiple organs simultaneously. It is important to identify the collective toxicity and mechanisms of damage caused by PM in various organs, considering that (1) the toxicity of PM may vary, and (2) the cellular responses to PM will differ depending on the tissue or organ. In particular, it is necessary to develop biomarkers that can evaluate the effects of PM, estimate the degree of damage, and identify therapeutic targets for treatment.

In this study, we formed 3D spheroids derived from eight human primary cell types of various tissue origins. We utilized 3D spheroids derived from nasal epithelial cells (NECs), bronchial/tracheal epithelial cells (B/TECs), lung fibroblasts (LFs), small airway epithelial cells (SAECs), microglia (M), astrocytes (As), dermal fibroblasts (DFs), and coronary artery endothelial cells (CAECs) to evaluate viability and proteomic changes after exposure to varying concentrations of DPM solutions for 24 and 48 h. To standardize the effects of DPM on these spheroids, standard reference material (SRM) 2975- from National Institute of Standards and Technology (NIST) was used as the source of DPM. Proteomic analysis using tandem mass tag (TMT) in combination with liquid chromatography- tandem mass spectrometry (LC-MS/MS) identified a total of 9,707 proteins, of which 128 exhibited statistically significant changes related to DPM concentrations. Among these, five proteins including apolipoprotein A-I (APOA1) exhibited statistically significant changes (*P*-value < 0.05) at the highest DPM concentrations, while 36 proteins, predominantly ribosomal proteins, demonstrated significant decreases even at lower DPM levels in the eight spheroid types. Canonical pathway analysis of the proteins that significantly increased in spheroids exposed to the highest DPM concentrations revealed common activation of acute phase response signaling, liver X receptor (LXR)/retinoid X receptor (RXR), and farnesoid X receptor (FXR)/RXR pathways across all eight spheroid types. Further validation using reverse transcription quantitative polymerase chain reaction (RT-qPCR) and Western blot analysis demonstrated that APOA1, a major protein component of high-density lipoprotein (HDL), was increased by DPM treatment via impaired protein degradation pathway. This study provides insights into the systemic impact of DPM and suggests that APOA1 may serve as a key marker for systemic responses to DPM exposure.

## Materials and methods

### Materials and reagents

DPM, which is SRM 2975 sourced from an industrial diesel-powered forklift, was purchased from National Institute of Standards and Technology (Gaithersburg, MD, USA). Antibodies against apolipoprotein H (APOH; mouse monoclonal, sc-515677, 1:200) and APOA1 (mouse monoclonal, sc-376818, 1:100) were purchased from Santa Cruz Biotechnology (Dallas, TX, USA). Antibody against glyceraldehyde-3-phosphate dehydrogenase (GAPDH; mouse monoclonal, MA5-15738, 1:5000) was purchased from Thermo Fisher Scientific (Waltham, MA, USA). Tripartite motif-containing protein 25 (TRIM25; rabbit monoclonal, ab167154, 1:2000) and β-actin antibodies (rabbit polyclonal, ab8227, 1:5000) were purchased from Abcam (Cambridge, MA, USA). Antibodies against sequestosome-1 (SQSTM1/p62; rabbit monoclonal, #8025, 1:1000) and microtubule-associated protein 1 light chain 3 beta (LC3B; rabbit monoclonal, #3868, 1:1000) were obtained from Cell Signaling Technology (Danvers, MA, USA). Horseradish peroxidase (HRP)-conjugated antibodies, goat anti-mouse IgG (SA001-500, 1:5000) and goat anti-rabbit IgG (SA002-500, 1:5000), were obtained from GeneDEPOT (Barker, TX, USA). GW6471, GSK2033, T0901317, MG-132, and cycloheximide (CHX) were purchased from Sigma-Aldrich (St. Louis, MO, USA).

### Cell culture

Human primary NECs (36010-12) and human primary M (37089-01) were obtained from Celprogen Inc. (Torrance, CA, USA), and were cultured in human nasal primary cell culture complete medium with serum and human M primary cell culture complete media with serum, respectively (Celprogen Inc.). Human primary LFs (CC-2512) and human primary As (CC-2565) were obtained from Lonza (Basel, Switzerland), and were cultured in fibroblast growth medium-2 BulletKit and astrocyte growth medium BulletKit (Lonza), respectively. Human primary B/TECs (PCS-300-010), human primary SAECs (PCS-301-010), human primary DFs (PCS-201-012), and human primary CAECs (PCS-100-020) were obtained from ATCC (Manassas, VA, USA). Human B/TECs and human SAECs were cultured in airway epithelial cell basal medium with bronchial epithelial cell growth kit (ATCC). Human DFs were cultured in fibroblast basal medium with fibroblast growth kit-low serum (ATCC), and human CAECs were maintained in vascular cell basal medium with endothelial cell growth kit-VEGF (ATCC). All types of the human primary cells were grown in a humidified incubator with 95% air and 5% CO_2_ at 37 ℃.

CCD-8Lu (CCL-201) and Hs68 (CRL-1635) cells obtained from ATCC were cultured in Eagle’s minimum essential medium medium with 10% fetal bovine serum. Cells were maintained in a humidified atmosphere with 5% CO_2_ at 37 °C and the medium was changed every 2 days after seeding. CCD-8Lu at passage < 10 and Hs68 at passage < 25 were used at 70% confluence for all the experiments.

### Preparation of DPM solutions

DPM stock solutions were made fresh at a concentration of 8 mg/mL in Dulbecco’s phosphate buffered saline (GenDEPOT, Barker, TX, USA) by vortexing for 60 min followed by sonication in a water bath sonicator (Branson 2510 Ultrasonic Cleaner, Brookfield, CT, USA) for 60 min. Then, the DPM solutions were diluted to 2X concentrations where X represents concentrations that were attempted to treat spheroids derived from human primary cells using each type of cell culture medium.

### Formation of spheroids and treatment of DPM solutions

Eight types of human primary cells were harvested at 60–90% confluence from 2D cultures and used at passages 5–15 depending on the cell type. Cells were seeded at 4.0 × 10^3^ to 1.2 × 10^4^ cells/well with 200 µL of the corresponding cell culture medium in ultra-low attachment 96 well plates (96-well, Nunclon Sphera-treated, U-shaped-bottom microplate, Thermo Fisher Scientific, Waltham, MA, USA) to produce spheroids. The plates were then centrifuged at 100 ~ 220 × *g* for 2 min at 35 °C using an Eppendorf 5810R centrifuge (Eppendorf, Hamburg, Germany). The formed spheroids were observed using 4X or 10X magnification objective on a Nikon Eclipse TE2000-S phase contrast microscope (Melville, NY, USA). After formation of spheroids, 100 µL of cell culture medium in each well was removed immediately prior to treatment of DPM solutions. Spheroids derived from NECs and M were treated with DPM solutions on the second day after spheroid formation, while spheroids from six other types of human primary cells were treated with DPM solutions the day after cell seeding. For treatment with DPM solutions, 100 µL of 2X concentrations of DPM solutions (ranging from 25 µg/mL to 3,200 µg/mL), depending on the spheroid types derived from eight kinds of human primary cells, were added to each well containing spheroids, resulting in final DPM concentrations of 12.5 µg/mL to 1,600 µg/mL. The DPM solutions were then briefly mixed without disturbing the spheroids. The DPM solutions were treated for 24 and 48 h, respectively.

### Measurement of viability of spheroids under treatment of DPM solutions

Viability of spheroids that were treated with different concentrations of DPM solutions was made using CellTiter-Glo 3D Cell Viability Assay (Promega, Madison, WI, USA). After transferring spheroids from each well to 1.5 mL microcentrifuge tubes, 200 µL of CellTiter-Glo 3D reagent was added to each tube and mixed vigorously to induce cell lysis followed by 30-min incubation at room temperature. Then, 200 µL of supernatant from each tube was transferred into an opaque-walled 96-well plate, and luminescence was measured using a microplate reader (BioTek Instruments, Inc., Winooski, VT, USA). Viability measurements were taken from three wells for each condition. The spheroid viability was calculated according to the following formula:

$$\:\text{S}\text{p}\text{h}\text{e}\text{r}\text{o}\text{i}\text{d}\:\text{v}\text{i}\text{a}\text{b}\text{i}\text{l}\text{i}\text{t}\text{y}\:\left({\%}\right)=\:\frac{\left(\text{l}\text{u}\text{m}\text{i}\text{n}\text{e}\text{s}\text{c}\text{e}\text{n}\text{c}\text{e}\:\text{s}\text{i}\text{g}\text{n}\text{a}\text{l}\:\text{o}\text{f}\:\text{t}\text{r}\text{e}\text{a}\text{t}\text{m}\text{e}\text{n}\text{t}\right)}{\left(\text{l}\text{u}\text{m}\text{i}\text{n}\text{e}\text{s}\text{c}\text{e}\text{n}\text{c}\text{e}\:\text{s}\text{i}\text{g}\text{n}\text{a}\text{l}\:\text{o}\text{f}\:\text{c}\text{o}\text{n}\text{t}\text{r}\text{o}\text{l}\right)}\:\times\:100{\%}\:$$

Data are expressed as the mean ± standard error of the mean (SEM) (*n* = 3 for each treatment). To evaluate the statistical significance of spheroid viability between untreated spheroids and those treated with increasing concentrations of DPM, statistical analyses were performed using one-way analysis of variance (ANOVA) for each time point individually, followed by Tukey’s multiple comparison test in GraphPad Prism software (version 10.2.3, GraphPad Software, Boston, MA, USA). Statistical significance was defined as *P < 0.05, **P < 0.01, ***P < 0.001, and ****P < 0.0001.

### 3-(4,5-dimethylthiazol-2-yl)−5-(3-carboxymethoxyphenyl)−2-(4-sulfophenyl)−2 H-tetrazolium (MTS) assay for 2D cell culture (Cell viability)

CCD-8Lu and Hs68 cells were plated at a density of 7,000 cells/cm² in a 60 mm dish. After overnight incubation, the cells were treated with varying concentrations of DPM for 24 h, and then the cell viability was measured using the MTS reagent (Thermo Fisher Scientific, MA, USA) according to the manufactures’ instructions. All the measurement data is from at least 3 independent replicative experiments, and quantitative data are expressed as the mean ± SEM. Statistical analyses were performed with Student’s *t*-test using Origin Pro 9.0 software (Originlab Corporation, MA, USA).

### In-solution digestion for proteomic analysis

For MS-based proteomic analysis, spheroids derived from eight types of human primary cells originating from various tissues were collected with the number of wells ranging from 40 to 65 for each cell type. These spheroids, including those treated with different concentrations of DPM solutions as well as untreated controls, were lysed in lysis buffer (7 M Urea, 2 M Thiourea, 1 mM ethylenediaminetetraacetic acid (EDTA), 150 mM NaCl, 50 mM tris(hydroxymethyl)aminomethane hydrochloride pH 7.5, and protease inhibitor cocktail (Roche Diagnostics, Mannheim, Germany) using a probe-type sonicator (Sonics & Materials, Newtown, CT, USA). After lysis, the samples were centrifuged to remove DPM particles, and protein concentrations were measured using a Bradford protein assay (Bio-Rad Laboratories, Inc. Hercules, CA, USA). Urea was then added to all samples to reach a final concentration of 8 M. The protein samples were reduced with 5 mM tris(2-carboxyethyl) phosphine hydrochloride and then alkylated with 10 mM iodoacetamide. The samples were treated with 25 mM ammonium bicarbonate to decrease the urea concentration to < 1 M and digested with lysyl endopeptidaseR (Lys-C, Fujifilm Wako Pure Chemical Corporation, Osaka, Japan) at an enzyme/substrate ratio of 1 milli–absorbance unit Lys-C per 50 µg total protein at 30 °C for 2 h^7^. Trypsin was then added to the samples (Promega, Madison, WI, USA) at a protease to substrate ratio of 1:50 (wt/wt) and incubated at 37 °C overnight. The resulting peptides were desalted using a Sep-Pak tC18 cartridge (Waters Corporation, Milford, MA, USA) and dried in a miVAC vacuum concentrator (Genevac Ltd., Ipswich, UK). After the peptide samples were resuspended in 100 mM triethyl ammonium bicarbonate (TEAB), the concentrations of peptides were measured using a quantitative colorimetric peptide assay kit (Thermo Fisher Scientific, Rockford, IL, USA).

### TMT labeling and basic pH reversed‑phase LC

After TMTpro 16plex label reagents (0.5 mg per vial, Thermo Fisher Scientific, Rockford, IL, USA, lot numbers VK309613, VL314803, and WD319901) were resuspended in 62 µL of anhydrous acetonitrile (ACN) per vial, 30 µL of each TMT labeling reagent was added to 21 µg of peptide samples for labeling. After the labeling reaction proceeded for 1 h at room temperature, 5 µL of 5% hydroxylamine in 100 mM TEAB was added and incubated for 15 min to stop the labeling reaction. Subsequently, Equal amounts (21 µg) of the 16 TMT-labeled peptide samples were combined and desalted using a Sep-Pak tC18 cartridge. The TMT-labeled peptide samples were resuspended in 10 mM ammonium formate (AF) and the peptide concentrations were determined using a quantitative colorimetric peptide assay kit. Then, 112.8 µg of the peptide sample was loaded onto an X-Bridge peptide BEH C18 column (4.6 mm i.d. × 250 mm length; pore size 130 Å; particle size 3.5 μm, Waters Corporation) and fractionated by basic pH reversed-phase liquid chromatography using an Agilent 1290 Infinity liquid chromatography (LC) system (Agilent Technology, Santa Clara, CA). Peptides were separated at a flow rate of 0.5 mL/min with the following gradient conditions: 0 min 100% buffer A (10 mM AF, pH 10) and 0% buffer B (10 mM AF, pH 10 in 90% ACN), 0–10 min 0–5% B, 10–48.5 min 5–40% B, 48.5–62.5 min 40–70% B, 62.5–72.5 min 70% B, 72.5–82.5 min 70 − 5% B, and 82.5–92.5 min 5% B. Fractionation was conducted by collecting 96 wells (1 well/0.8 min, Restek corporation, Bellefonte, PA, USA) during the chromatographic run (10–82.5 min). The resultant 96 fractions were pooled to 12 concatenated fractions, dried, and subsequently resuspended in 23.5 µL 0.4% acetic acid.

### LC-MS/MS analysis

2.6 µg of the fractionated peptide samples were injected onto a trap column (2 cm x 75 μm inner diameter (i.d.), 100 Å, 3 μm) and separated on a reversed-phase Acclaim PepMap RSLC C18 column (50 cm × 75 μm i.d., 100 Å, 2 μm) using an UltiMate 3000 RSLCnano System (Thermo Fisher Scientific, Bremen, Germany). The column temperature was constantly set to 50 ℃ by using column heater. The operating flow rate was 300 nL/min with the following gradient conditions: 0 min 95% buffer A (100% water with 0.1% formic acid) and 5% buffer B (100% ACN with 0.1% formic acid), 0–4 min 5% B, 4–14 min 5–10% B, 14–97 min 10–25% B, 97–99 min 25–28% B, 99–102 min 28–40% B, 102–105 min 40–90% B, 105–106 min 90% B, 106–110 min 90 − 5% B, and 110–120 min 5 − 0%B. The nano ultra-high-performance liquid chromatography system was coupled to an Orbitrap Eclipse Tribrid Mass Spectrometer (Thermo Fisher Scientific, Bremen, Germany). MS1 data were collected using the Orbitrap (resolution 120,000; scan range 375–1,575 *m/z*; maximum injection time 247 ms; automatic gain control (AGC) 1 × 10^6^). Determined charge states between 2 and 8 were used for MS2 analysis and dynamic exclusion was set to 30 s. We used data-dependent ‘top 20’ MS2 scans that consisted of higher energy collision dissociation with the following parameters: resolution 30,000; isolation window in 1.4 *m/z*; normalized collision energy 35%; maximum injection time 55 ms; AGC 1 × 10^5^. At a resolution of 30,000, the MS2 scans provided sufficient mass accuracy and resolving power to clearly distinguish all 16 TMTpro reporter ions, including closely spaced channels such as 127 N and 127 C. Each of the peptide samples were run in triplicate using LC-MS/MS analysis.

### Data processing for protein identification, quantification, and pathway analysis

MS raw files were searched against the SwissProt human database (January 2021) with 20,343 entries using Proteome Discoverer software (version 2.4, Thermo Fisher Scientific). The search criteria were set to a mass tolerance of 10 parts per million (ppm) for MS data and 0.02 Da for MS/MS data with fixed modifications of carbamidomethylation of cysteine (+ 57.021 Da) and TMT on lysine residues and peptide N termini (+ 304.207 Da) and variable modification of methionine oxidation (+ 15.995 Da). The false discovery rate (FDR) was set at 0.01 for identification of peptides and proteins. All the proteins were identified by one or more unique peptides. Reporter ion quantification was performed with a 20 ppm mass tolerance and signal-to-noise ratio values of reporter ions were used for quantification of peptides. Reporter ion isotopic distribution correction was first pursued in order to correct the isotopic impurities of the TMTpro reagents before calculating signal-to-noise ratio values. That is, the reporter ion isotopic distributions obtained from the product data sheets (VK309613/VL314803/WD319901) of the TMTpro reagents were used as isotope correction factors in TMTpro 16plex method template in Proteome Discoverer software (version 2.4)^8^. Only spectra with an average reporter signal-to-noise ratio threshold of ≥ 10 across all 16 TMTpro 16plex channels were considered for quantification. The signal-to-noise ratio values for each reporter ion channel were summed across all quantified proteins and then normalized so that the summed signal-to-noise ratio values were equal across all 16 channels. These normalized values are referred to as normalized abundance values. Proteins with quantifiable values in at least two out of three LC-MS/MS runs were subjected to subsequent statistical analysis. The normalized signal-to-noise ratio values were first log-transformed and missing values were then replaced using values computed from the normal distribution with a width of 0.3 and a downshift of 1.8. A two-way analysis ANOVA was employed to analyze the proteomic data, aiming to identify proteins showing statistically significant changes in response to varying concentrations of DPM solutions and different exposure durations using Perseus software (1.6.14.0)^9^. The interaction analysis for proteins exhibiting significant changes (> 1.5 or < 0.67) in spheroids exposed to the highest DPM concentrations compared to unexposed spheroids at 48 h across all eight spheroid types, with statistical significance (*P*-value < 0.05) related to DPM concentrations as determined by two-way ANOVA analysis, was based on the STRING version 12.0 database (https://string-db.org/) using a high confidence score (0.8). The results were visualized using Cytoscape 3.10.2 where the nodes in the network were colored based on the log_2_ fold-change values obtained from NEC-S dataset. Ingenuity pathway analysis (IPA) software (data version 73620684; QIAGEN, Redwood City, CA, USA) was used to explore the signaling pathways related to proteins that showed significant increases (> 1.5-fold) in the spheroids exposed to the highest DPM concentrations compared to unexposed spheroids at 48 h, among the proteins with statistical significance (*P*-value < 0.05) from the two-way ANOVA based on DPM concentrations. The IPA Z-score algorithm was used to predict activation states of canonical pathways based on the directionality of expression changes. A Z-score ≥ 2 was interpreted as significant activation, while a Z-score ≤–2 was considered significant inhibition^10^. If no Z-score was assigned, the activation state of the pathway could not be predicted due to insufficient directional information.

### Western blot analysis

3D spheroid colonies were collected from U-bottom 96 well plate, washed with ice-cold phosphate-buffered saline (Welgene, Gyeongsan, Korea), and lysed with either 7 M urea that contained with 2 M thiourea, 1 mM EDTA, 150 mM NaCl, or 50 mM Tris-buffered radioimmunoprecipitation assay buffer (Biosesang, Youngin, Korea) with phosphatase inhibitor (GenDEPOT) and protease inhibitor cocktail (Sigma-Aldrich). All lysed samples were sufficiently centrifuged to remove DPM particles and cell debris. Equal amounts of total protein determined by the Bicinchoninic acid protein assay (Thermo Fisher Scientific) were diluted using sodium dodecyl sulfate (SDS) sample buffer (Bio-solution, Gyeonggi-do, Korea), boiled for 3 to 5 min, and 20 µg of each samples were loaded to 10% SDS-poly acrylamide electrophoresis gels. The gels were then transferred onto polyvinylidene fluoride membranes (Amersham Biosciences, NJ, USA) for blotting. Each membrane was blocked with 5% nonfat dry milk in tris-buffered saline containing 0.1% Tween-20 and incubated with primary antibodies overnight at 4 °C. Blots were developed with a HRP-conjugated secondary antibody for 1 h at room temperature, followed by visualization using WSE-7120 EzWestLumi plus HRP substrate (ATTO, Tokyo, Japan) and ChemiDoc MP (Bio-Rad Laboratories, CA, USA). All the measurement data is from at least 3 independent replicative experiments, and quantitative data are expressed as the mean ± SEM. Statistical analyses were performed with Student’s *t*-test using Origin Pro 9.0 software.

### Ribonucleic acid (RNA) interference

Scrambled small interfering RNAs (siRNAs) for control measurements and TRIM25 were purchased from Bionner Inc. (Daejeon, Korea) and Sigma-Aldrich (St. Louis, MO, USA), respectively. The following siRNAs were used: Scrambled siRNA (No. SN-1003); TRIM25-directed siRNA (F: 5’-CCCUGAGGCACAAACUAACTT-3’, R: 5’-GUUAGUUUGUGCCUCAGGGTG-3’)^11^. The 50 nM of siRNAs for TRIM25 was introduced into cells in Opti-MEM (GIBCO, Grand Island, N.Y.) using Lipofectamine RNAiMAX reagent (Invitrogen) following the manufacturer’s instructions. Scrambled siRNAs were used for all of the control measurements.

### RNA isolation and RT-qPCR

Total RNA was isolated using GeneJET RNA Purification Kit (Thermo Fisher Scientific) and then cDNA was synthesized using TOPscript™ cDNA Synthesis Kit (Enzynomics, Daejeon, Korea) according to the manufacturer’s instructions. RT-qPCR was performed using AccuPower^®^ 2X GreenStar™ qPCR Master Mix (Bioneer, Daejeon, Korea), 20 ng of cDNA mixture, and 5 pmol of each primer (Table S1), and simultaneously analyzed using QuantStudio 1 Real-Time PCR system (Thermo Fisher Scientific). The RT-qPCR amplification conditions were as follows: pre-incubation at 50 °C for 2 min, pre-denaturation at 95 °C for 10 min and 40 cycles of amplification (95 °C for 15 s and 60 °C for 1 min). The expression level of target gene was normalized to GAPDH expression and the messenger RNA (mRNA) expression was compared according to the 2^−ΔΔCT^ method. All the measurement data is from at least 3 independent replicative experiments, and quantitative data are expressed as the mean ± SEM. Statistical analyses were performed with Student’s *t*-test using Origin Pro 9.0 software.

## Results and discussion

### Viability assessment of 3D spheroids from eight primary cell types treated with DPM solutions

To form spheroids from eight types of primary cells derived from various human tissue origins, we first seeded different cell numbers of each cell type in U-bottomed ultra-low attachment plates. After evaluating spheroid formation under each seeding condition using phase contrast microscopy, we selected the optimal cell number for each cell type for subsequent experiments. One day after seeding, spheroids were visible although their sizes and compactness varied among the different cell types (See Fig. S1).

**Fig. 1:**
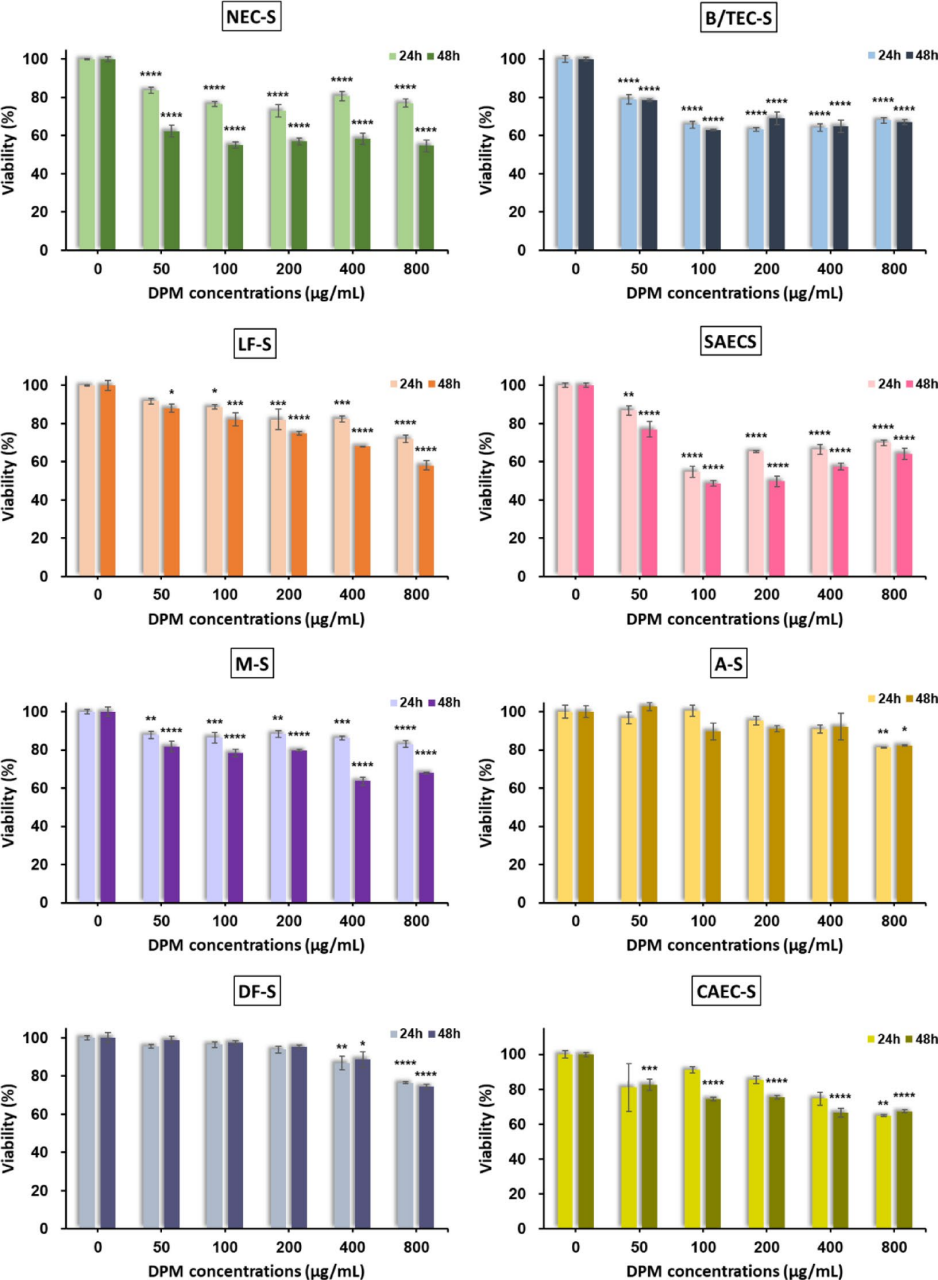
Viability of spheroids derived from eight primary cell types treated with DPM concentrations of 50, 100, 200, 400, and 800 µg/mL. Data are expressed as the mean ± SEM (*n* = 3 for each treatment). Statistical significance was evaluated using one-way ANOVA for each time point, followed by Tukey’s multiple comparison test. Data with *P < 0.05, **P < 0.01, ***P < 0.001, and ****P < 0.0001 were considered statistically significant.

After the optimal seeding conditions for each spheroid type were determined, the spheroids were exposed to increasing concentrations of DPM solutions for 24 and 48 h, respectively, and their viability was assessed. Figure 1 shows the viability of eight spheroid types exposed to DPM concentrations of 50, 100, 200, 400, and 800 µg/mL. Additionally, Fig. S2 illustrates the viability of each spheroid type at their respective maximum DPM concentrations (800 µg/mL or 1600 µg/mL), determined from initial viability tests. Overall, a general trend of decreased spheroid viability with increasing DPM concentrations was observed, although the extent of reduction showed noticeable variations among the eight spheroid types. Specifically, six of the eight spheroid types (NEC-derived spheroids (NEC-S), B/TEC-derived spheroids (B/TEC-S), LF-derived spheroids (LF-S), SAEC- derived spheroids (SAEC-S), M-derived spheroids (M-S), and CAEC-derived spheroids (CAEC-S)) exhibited significant decreases in viability at DPM concentrations of 50 µg/mL and above at either 24–48 h. In contrast, astrocyte-derived spheroids (A-S) showed significant reductions in viability at 800 µg/mL at both 24 and 48 h, while dermal fibroblast-derived spheroids (DF-S) exhibited significant decreases at 400 µg/mL. Notably, NEC-S showed significant reductions in viability at 12.5 µg/mL for both 24 and 48 h, while M-S exhibited a significant reduction at 12.5 µg/mL only at 48 h. Additionally, B/TEC-S demonstrated a significant decrease in viability at 25 µg/mL. Despite these dose-dependent effects, viability profiles were generally comparable between 24 h and 48 h across all spheroid types, indicating that DPM-induced cytotoxicity was largely initiated within the first 24 h and showed limited further progression with prolonged exposure.

### LC-MS/MS analysis of eight spheroid types exposed to DPM solutions

Following the viability assessment, LC-MS/MS analysis was pursued to investigate the proteomic changes in the eight types of spheroids exposed to varying concentrations of DPM solutions. The exposure concentrations for each spheroid type were: NEC-S, B/TEC-S, and SAEC-S were exposed to 25, 50, 100, 200, and 400 µg/mL; LF-S were exposed to 25, 50, 100, 200, 400, and 800 µg/mL; M-S were treated with 12.5, 25, 100, 400, and 800 µg/mL; A-S and DF-S were exposed to 200, 400, 800, and 1600 µg/mL; and CAEC-S were subjected to 50, 100, 200, 400, and 800 µg/mL. These concentrations were selected based on spheroid viability results to ensure that they encompassed similar ranges of viability changes across each spheroid type.

TMT labeling coupled with LC-MS/MS analysis of spheroids derived from eight primary cell types exposed to different concentrations of DPM solutions resulted in the identification of a total of 9,707 proteins. The number of proteins identified for each spheroid type was as follows: 6,602 for NEC-S, 6,805 for B/TEC-S, 6,288 for LF-S, 7,255 for SAEC-S, 7,337 for M-S, 6,985 for A-S, 5,807 for DF-S, and 5,870 for CAEC-S (see Table S2-S9). The identification status of these 9,707 proteins across the eight spheroid types is detailed in Table S10. An UpSet plot (Fig. S3) illustrates the overlap of these proteins among spheroid types, revealing that 3,932 proteins were commonly identified across all eight spheroid types, with additional overlaps observed among different spheroid combinations.

Of the identified proteins, only those with quantifiable values in at least two out of three LC-MS/MS runs for each condition were subjected to a two-way ANOVA to identify proteins that showed statistically significant changes in response to different DPM concentrations and exposure durations. The quantifiable proteins for each spheroid type were: 6,194 for NEC-S, 6,214 for B/TEC-S, 5,780 for LF-S, 6,748 for SAEC-S, 6,888 for M-S, 6,391 for A-S, 5,113 for DF-S, and 5,148 for CAEC-S. All MS2 spectra were acquired at a resolution of 30,000, which was sufficient to fully resolve all 16 TMTpro reporter ions, including closely spaced channels such as 127 N and 127 C, thereby supporting the reliability of MS2-based quantification used in subsequent analyses.

Detailed results of the statistical analysis are provided in the supplementary tables (Tables S11-S18). Table 1 summarizes the two-way ANOVA results, including the number of proteins that exhibited statistical significance (*P*-value < 0.05) based on DPM concentrations, exposure durations, and their interaction among the eight spheroid types. Venn diagram analysis of these statistically significant proteins across these eight spheroid types revealed that 128 proteins were statistically significant across all spheroids based on DPM concentrations, five proteins based on exposure durations, and one protein based on the interaction between these factors (Table S19). The greater number of statistically significant proteins associated with DPM concentrations across all eight spheroid types, as determined by the two-way ANOVA, suggests that DPM concentration is a more critical determinant of protein abundances compared to exposure durations or their interaction.

**Table 1:** Summary of two-way ANOVA results and the number of proteins showing statistically significant changes in response to DPM exposure in eight spheroid types.

Spheroids	Number of proteins with quantifiable information	*P*-value_ time points^a^ <0.05	*P*-value_DPM concentrations^b^ < 0.05	*P*-value_ interaction^c^ < 0.05
NEC-S	6,194	3,107	646	215
B/TEC-S	6,214	839	3,049	435
LF-S	5,780	1,445	2,662	507
SAEC-S	6,748	920	1,751	307
M-S	6,888	891	2,311	259
A-S	6,391	251	3,143	153
DF-S	5,113	1,138	2,651	911
CAEC-S	5,148	2,339	2,199	524

^*^NEC-S, B/TEC-S, LF-S, SAEC-S, M-S, A-S, DF-S, and CAEC-S represent nasal epithelial cell-derived spheroids, bronchial/tracheal epithelial cell-derived spheroids, lung fibroblast-derived spheroids, small airway epithelial cell-derived spheroids, microglia-derived spheroids, astrocyte-derived spheroids, dermal fibroblast-derived spheroids, and coronary artery endothelial cell-derived spheroids, respectively.

^a^*P*-value was obtained from two-way ANOVA comparison of the log_2_(normalized signal-to-noise values) and indicates the statistical significance between 24 and 48 h.

^b^*P*-value was derived from two way ANOVA comparison of the log_2_(normalized signal-to-noise values) and reflects the statistical significance among different diesel particulate matter (DPM) concentrations.

^c^*P*-value was obtained from two way ANOVA comparison of the log_2_(normalized signal-to-noise values) and indicates the statistical significance interaction between time points and DPM concentrations.

To further explore how DPM concentrations impact protein abundance, hierarchical clustering analysis was performed on the proteins showing statistically significant changes (*P*-value < 0.05) in response to DPM concentrations for each spheroid type (Fig. S4). The heat maps generated from this analysis revealed distinct patterns of protein abundance for each spheroid type. In B/TEC-S, SAEC-S, M-S, DF-S, and CAEC-S, proteins exhibited tighter clustering, particularly at elevated DPM concentrations, indicating a more uniform response to DPM exposure. On the other hand, NEC-S, LF-S, and A-S displayed more dispersed clustering, with broader variability in protein abundance patterns with increasing DPM concentrations. Hierarchical clustering was also performed on the 128 proteins that exhibited statistical significance (*P*-value < 0.05) across all eight spheroid types with respect to DPM concentrations (Fig. S5). The resulting heat map revealed more distinct clustering patterns in each spheroid type, with most proteins tightly clustered at both elevated DPM concentrations and unexposed conditions.

**Fig. 2:**
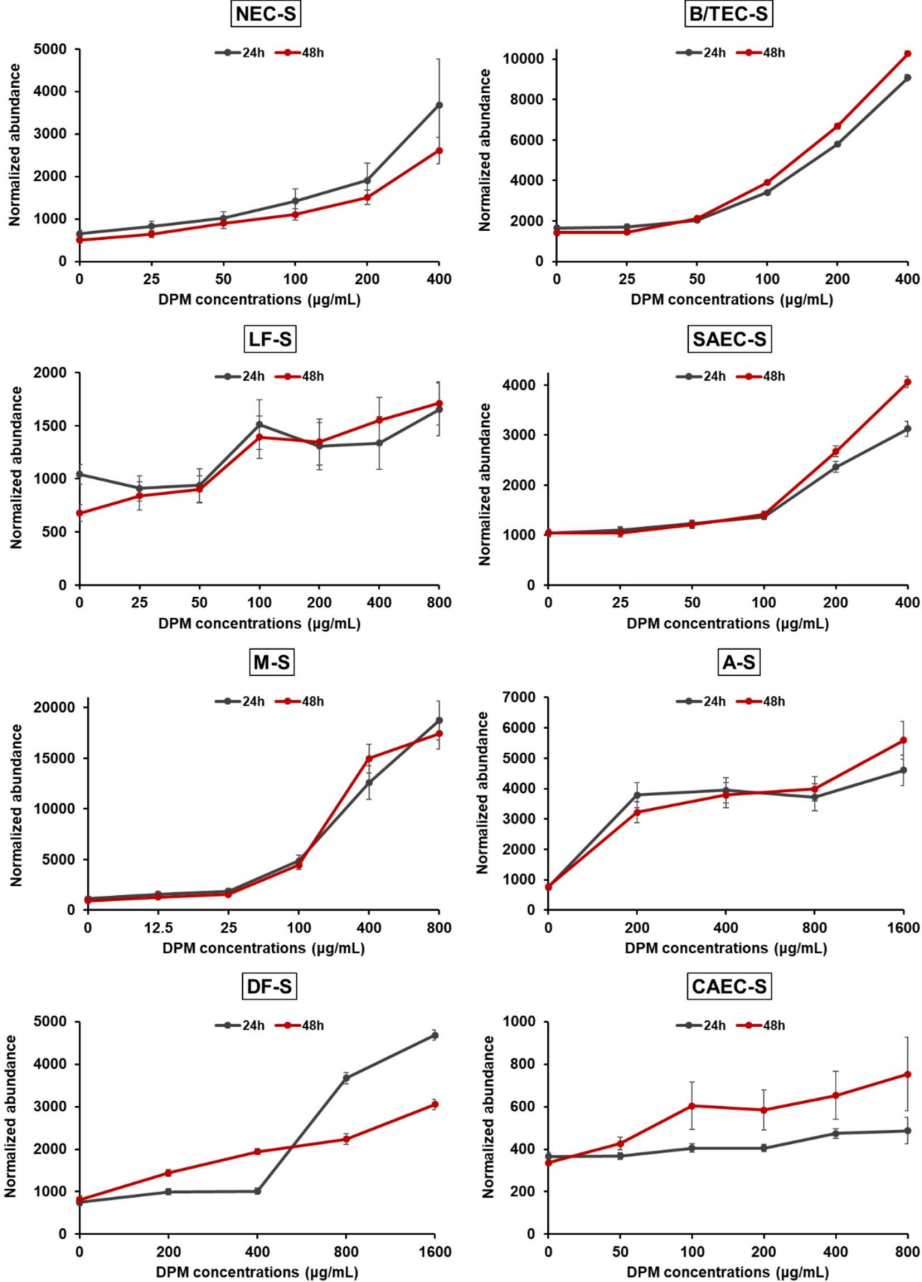
Abundance levels of APOA1 in the eight types of spheroids exposed to different DPM concentrations at 24 and 48 h. Data were obtained from three LC-MS/MS runs and are expressed as the mean ± SEM. The abundance of APOA1 increases with higher DPM concentrations at both time points.

We then examined the abundance changes of the 128 statistically significant proteins (*P*-value < 0.05) across all eight spheroid types with respect to DPM concentrations comparing unexposed spheroids to those exposed to the highest DPM concentrations at 48 h for each spheroid type. The 48 h time point was chosen over 24 h because prolonged exposure is expected to produce more substantial alterations in protein levels, revealing more pronounced effects of DPM. As a result, five proteins, including APOA1 (P02647) showed significant increases (> 1.5-fold) at the highest DPM concentrations, whereas 36 proteins showed significant decreases (< 1.5-fold) across all eight spheroid types (Table S20 with fold changes at both 24 and 48 h). Specifically, for APOA1, fold changes and corresponding *P*-values at 48 h were as follows: NEC-S (5.22; *P*-value = 3.80 × 10⁻⁸), B/TEC-S (7.20; *P*-value = 1.33 × 10⁻²²), LF-S (2.52; *P*-value = 5.33 × 10⁻⁵), SAEC-S (3.89; *P*-value = 1.59 × 10⁻¹⁷), M-S (19.18; *P*-value = 2.33 × 10⁻¹⁹), A-S (7.28; *P*-value = 2.78 × 10⁻¹²), DF-S (3.76; *P*-value = 3.02 × 10⁻¹⁶), and CAEC-S (2.24; *P*-value = 7.62 × 10⁻³) (Table S19a and S20). Notably, most of the proteins exhibiting significant decreases (< 1.5-fold) were ribosomal proteins. Figure 2 illustrates the abundance levels of APOA1 at different DPM concentrations over 24 and 48 h, demonstrating that its levels increased with increasing DPM concentrations. Fig. S6 further shows four proteins (beta-2-glycoprotein 1 (APOH), kininogen-1 (KNG1), apolipoprotein C-III (APOC3), and pigment epithelium-derived factor (SERPINF1)) that were commonly increased by more than 1.5-fold in all eight spheroid types, and three proteins (40 S ribosomal protein S24 (RPS24), 60 S ribosomal protein L34 (RPL34), 60 S ribosomal protein L37 (RPL37) among the 36 proteins that were decreased by more than 1.5-fold across all eight spheroids at the highest DPM concentration at 48 h. Notably, these proteins exhibited comparable abundance patterns at 24 h and 48 h, suggesting that the DPM-induced proteomic response was rapidly initiated and remained stable throughout the exposure period. KNG1, alongside APOA1, exhibited a consistent increase in abundance with rising DPM concentrations at both 24 and 48 h, whereas APOH, APOC3, and SERPINF1 peaked at lower DPM concentrations in some spheroid types and tended to decrease as DPM concentrations increased. These biphasic patterns may reflect dose-dependent regulation of cellular stress response pathways, potentially consistent with hormetic behavior, where moderate stress activates protective mechanisms and excessive stress leads to cellular dysfunction^12^. APOH is involved in coagulation, lipid transport, and immune regulation^13^. Its increased abundance at intermediate concentrations may indicate an acute-phase or compensatory immune response, whereas persistent oxidative or inflammatory stress at higher exposures may impair these physiological functions or promote protein degradation. APOC3, a key modulator of triglyceride-rich lipoproteins, is known to be downregulated by inflammatory stimuli, including tumor necrosis factor-alpha (TNF-α), via the nuclear factor kappa-light-chain-enhancer of activated B cells (NF-κB) pathway^14^. Its increased abundance at moderate DPM concentrations may reflect lipid remodeling or a compensatory response to mild stress, while its decline at higher exposures could reflect inflammation-mediated suppression. SERPINF1 is a multifunctional protein with anti-angiogenic, anti-inflammatory, and antioxidant properties. Its expression increases under moderate oxidative stress as part of a cytoprotective response^15,16^. The biphasic pattern observed for SERPINF1 may therefore reflect transient protective response, followed by translational inhibition or broader stress-induced suppression at higher concentrations. Although biphasic expression patterns for APOH, APOC3, or SERPINF1 under environmental stress have not been directly reported in previous studies, similar biphasic responses have also been reported in both in vivo and cellular models. For example, Husain et al. observed early induction, suppression, and later reactivation of inflammatory gene expression in mouse lungs following carbon black nanoparticle exposure^17^. Escobar et al. observed biphasic expression of p62 in peripheral blood mononuclear cells following acute resistance exercise, a physiological stressor, where p62 levels initially decreased and later increased, suggesting a temporally regulated stress response^18^. These findings collectively support the biological plausibility of the biphasic expression patterns observed in our study. However, since these trends were not consistently observed across all eight spheroid types, this variability may reflect cell type–specific differences in the response to DPM exposure. On the other hand, the three ribosomal proteins (RPS24, RPL34, and RPS37) showed a sharp decrease in abundance at lower DPM concentrations, with these reductions maintained over both 24 and 48 h. In particular, RPL37 showed a sharp and early reduction at the lowest tested DPM concentrations across all eight spheroid types. Importantly, this marked decrease occurred despite relatively high spheroid viability at the corresponding concentrations (Fig. S2), suggesting that the response of RPL37 may not simply reflect cytotoxicity, but rather an early regulatory adaptation to DPM-induced stress. DPM contains redox-active components, such as heavy metals and organic compounds, that can induce the generation of reactive oxygen species^19^. Ribosomal proteins, which are typically highly expressed, are particularly sensitive to cellular stress and may be downregulated early as part of a protective response. The accumulation of ROS can damage DNA, RNA, and proteins, thereby disrupting transcription and translation^20^. Additionally, DPM induces inflammatory responses, leading to the secretion of cytokines such as TNF-α, interleukin-1 beta, and interleukin-6 ^21^. These cytokines promote translational repression and can negatively regulate the transcription of ribosomal components^22^. Taken together, these stress-mediated pathways may contribute to the selective and early downregulation of ribosomal proteins like RPL37 under low-dose DPM exposure conditions. Although there is limited direct evidence that RPL37 is more sensitive to environmental stress than other ribosomal proteins, previous studies have shown that it can participate in ribosomal stress responses by modulating the mouse double minute 2 homolog (MDM2)–tumor protein p53 (p53) signaling pathway, where it suppresses MDM2 activity and promotes p53 stabilization^23^. This reported function raises the possibility that RPL37 may be among the ribosomal proteins more susceptible to early stress-mediated regulation. While our study did not include concentrations below the lowest tested dose, more gradual response patterns were observed in other ribosomal proteins, such as RPL34 and RPS24, across a broader concentration range. These differences suggest that early responses to DPM exposure may vary depending on the specific ribosomal protein, and that in the case of RPL37, regulatory changes may have been initiated at concentrations lower than those tested in our study.

While TMT-based quantification in this study was performed using MS2-based acquisition due to its high throughput and broad proteome coverage, this approach is inherently susceptible to ratio compression due to co-isolation interference^24,25^. Real-Time Search MS3 (RTS-MS3) has been shown to address these limitations by improving quantification accuracy while maintaining proteome coverage comparable to MS2-based methods, owing to its increased acquisition efficiency^26^. Although not applied in this study, RTS-MS3 represents a promising option for future large-scale proteomic experiments requiring higher quantitative precision.

**Fig. 3:**
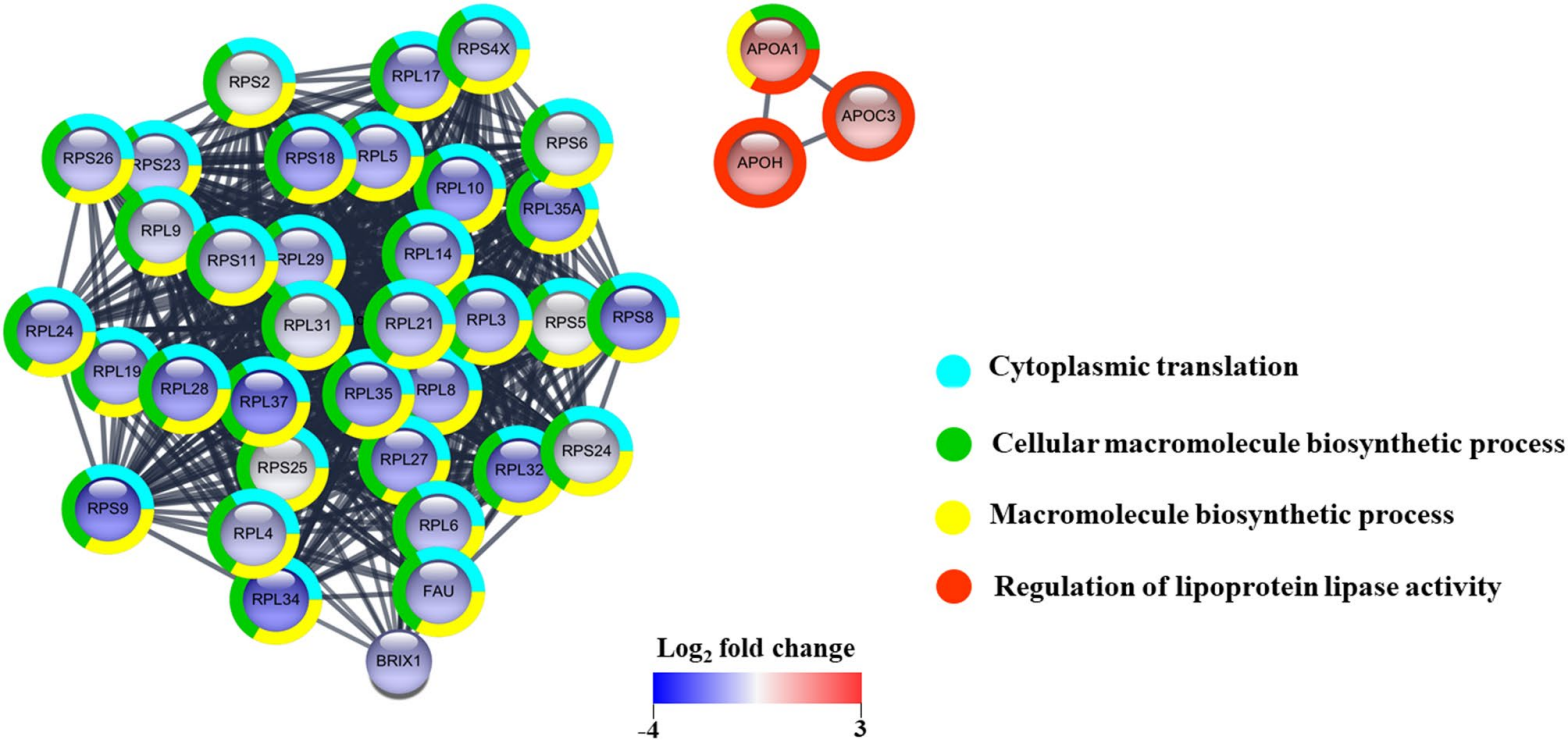
Interaction of 41 proteins that showed significant changes (> 1.5 or < 0.67) in the spheroids exposed to the highest DPM concentrations compared to unexposed spheroids at 48 h across all eight spheroid types, with statistical significance (*P*-value < 0.05) related to DPM concentrations as determined by two-way ANOVA analysis. Of these, 38 proteins that were connected in the network are visualized. The analysis was done using protein interaction information from the STRING database and visualized using Cytoscape. Each node represents a protein that is colored on the log_2_ fold change values in the spheroids exposed to the highest DPM concentrations compared to unexposed spheroids at 48 h based on NEC-S dataset, with red representing upregulated proteins and blue indicating downregulated proteins. Additionally, the edges of the nodes are represented in a donut shape, with the color of the edges depicting the corresponding GO BP terms.

Next, we performed a protein-protein interaction network analysis on the 41 proteins that showed statistically significant changes (> 1.5 or < 0.67) across all eight spheroid types in response to the highest concentrations of DPM treatment (Fig. 3). The interaction analysis revealed significant enrichment of gene ontology (GO) biological process (BP) terms, with a FDR < 0.05 (Table S21). The top three enriched biological processes (cytoplasmic translation, cellular macromolecule biosynthetic process, and macromolecule biosynthetic process) are primarily associated with proteins that exhibited significant decreases in response to DPM treatment. This suggests that DPM exposure may impair the synthesis of proteins and macromolecules, which are crucial for maintaining the structure and functions of spheroids. On the other hand, the term related to the regulation of lipoprotein lipase activity is specifically linked to the upregulated proteins, APOA1, APOH, and APOC3. This indicates that the lipid transport and metabolism processes associated with these proteins may be affected by DPM treatment.

### Canonical pathway analysis

IPA software was used to analyze proteins that exhibited significant increases (> 1.5-fold) in spheroids exposed to the highest DPM concentrations compared to unexposed spheroids at 48 h, aiming to explore related signaling pathways. The top five canonical pathways for each spheroid type are listed in Table 2. Among these pathways, acute phase response signaling and LXR/RXR activation were commonly observed in all eight spheroids. FXR/RXR activation was found in seven spheroid types except A-S; however, it ranked as the seventh canonical pathway (*P*-value: 2.51 × 10^−8^) in the A-S, indicating that these three pathways are linked to the proteins exhibiting significant increases in response to the highest concentrations of DPM.

**Table 2:** Top five canonical pathways obtained from IPA for proteins exhibiting significant increases (> 1.5-fold) at the highest DPM concentrations compared to the unexposed spheroids.

NEC-S			B/TEC-S		
Top canonical pathways	*P*-value	Z-score	Top canonical pathways	*P*-value	Z-score
LXR/RXR activation**	7.31E-34	4.2	LXR/RXR activation**	3.50E-27	5.376
FXR/RXR activation*	1.16E-33	–	FXR/RXR activation*	7.22E-27	–
Acute phase response signaling**	5.32E-29	1.667	Acute phase response signaling**	5.88E-19	2.065
Coagulation system	4.55E-15	−0.632	Atherosclerosis signaling	3.06E-12	–
Atherosclerosis signaling	1.69E-14	–	SNARE signaling pathway	9.40E-12	4.315

LF-S			SAEC-S		
Top canonical pathways	*P*-value	Z-score	Top canonical pathways	*P*-value	Z-score
LXR/RXR activation**	2.09E-15	3.962	FXR/RXR activation*	3.78E-37	–
Acute phase response signaling**	1.03E-12	1.732	LXR/RXR activation**	1.91E-32	5.292
FXR/RXR activation*	1.47E-11	–	Acute phase response signaling**	7.78E-26	1.387
Hepatic fibrosis/Hepatic stellate cell activation	7.67E-11	–	Atherosclerosis signaling	5.30E-11	–
Clathrin-mediated endocytosis signaling	2.65E-09	–	Clathrin-mediated endocytosis signaling	8.91E-08	–

M-S			A-S		
Top canonical pathways	*P*-value	Z-score	Top canonical pathways	*P*-value	Z-score
FXR/RXR activation*	1.53E-27	–	Acute phase response signaling**	2.18E-12	2.84
Acute phase response signaling**	1.52E-25	1.941	LXR/RXR activation**	1.04E-10	3.545
LXR/RXR activation**	3.80E-25	4.914	GP6 signaling pathway	1.24E-10	4.379
Complement system	3.21E-16	1	Hepatic fibrosis/Hepatic stellate cell activation	1.28E-10	–
Coagulation system	7.67E-15	0.277	Pulmonary fibrosis idiopathic signaling pathway	2.31E-10	6.008

DF-S			CAEC-S		
Top canonical pathways	*P*-value	Z-score	Top canonical pathways	*P*-value	Z-score
FXR/RXR activation*	3.10E-13	–	LXR/RXR activation**	1.80E-15	3.674
LXR/RXR activation**	1.19E-11	4.69	FXR/RXR activation*	1.69E-12	–
Acute phase response signaling**	1.30E-11	1.606	Acute phase response signaling**	5.43E-12	2.309
Clathrin-mediated endocytosis signaling	9.58E-10	–	Hepatic fibrosis/Hepatic stellate cell activation	6.67E-11	–
Pulmonary fibrosis idiopathic signaling pathway	8.14E-09	4.95	Clathrin-mediated endocytosis signaling	1.15E-08	–

§IPA was conducted on the proteins showing significant increases (> 1.5-fold) at 48 h in spheroids exposed to the highest DPM concentrations compared to unexposed spheroids, among those proteins identified as statistically significant by two-way ANOVA across the eight spheroid types.

†*P*-value is displayed in E notation: aEb indicates a value of a × 10^b^.

¥Z-score reflects the predicted activation state of a pathway. A Z-score ≥ 2 suggests significant activation, while a Z-score ≤ − 2 suggests significant inhibition. A missing Z-score (–) indicates that no activation state could be predicted due to insufficient directional information.

£ **indicates the pathways recognized as the top five signaling pathways across all eight spheroid types, while * denotes pathways that are common to seven of the spheroid types based on IPA analysis.

Acute phase response signaling, one of the key canonical pathways in response to the highest DPM concentrations across all spheroid types, is activated in response to a local or systemic disturbance caused by infection, tissue injury, trauma, or immunological disorders. Pro-inflammatory cytokines are released, leading to systemic reactions including fever, decreased leukocyte numbers in blood, increased levels of adrenocorticotrophic hormone, activation of blood coagulation, and changes in several plasma protein concentrations (for review, see^27^). Concentrations of ceruloplasmin, complement component 3, haptoglobin, fibrinogen, α-globulins, C-reactive protein (CRP), serum amyloid A (SAA) are increased. CRP and SAA can bind to microoganisms and lipopolysaccharides (LPS), respectively, thus preventing infection-induced responses^28^. In fact, it has been reported that PM is associated with the acute phase response in cardiovascular disease, pulmonary inflammation, and lung injury^21,29,30^. Although activation was confidently predicted (Z-score ≥ 2) in only three spheroids (B/TEC-S: 2.065; A-S: 2.84; CAEC-S: 2.309), the remaining five exhibited positive but sub-threshold Z-scores (1.387–1.941), suggesting a general trend toward activation in response to DPM exposure. Since this pathway ranked among the top five canonical pathways in all eight spheroid types based on 48 h proteomic data, and given that protein abundance patterns remained largely stable between 24 h and 48 h (Fig. 2 and Fig. S6), it is likely that activation occurred early and was sustained throughout the exposure period. These findings suggest that the cellular response to DPM involved early engagement of stress pathways with limited changes thereafter, which may explain the minimal differences observed between the two time points.

On the other hand, the LXR/RXR pathway is activated by the binding of cholesterol derivatives to LXR and regulates genes involved in cholesterol homeostasis, glucose and lipid metabolism, inflammation, and innate immunity^31^. Specifically, LXR/RXR has been shown to mediate anti-inflammatory activity by inhibiting the NF-κB, activator protein 1, or signal transducer and activator of transcription 1 pathways, playing critical roles in innate immune response and immune cell function^31,32^. Therefore, LXR/RXR becomes a potential therapeutic target for various diseases, including atherosclerosis, autoimmune disorders, and neurodegenerative diseases by modulating its activity with chemical ligands^33^. In our study, the LXR/RXR pathway showed consistent activation (Z-score ≥ 2) across all eight spheroid types, indicating robust and uniform activation of this pathway in response to DPM exposure. Similarly, FXR/RXR pathway is activated when bile acids bind to FXR and regulates genes involved in bile acid, glucose, lipid, and triglyceride metabolism^34^. FXR is expressed in several types of cells of innate immunity and responsible for regulation of TNF-α and interleukin-12 in dendritic cells, osteopontin in Natural killer T cells, and macrophage polarization, suggesting a potential role for liver and intestinal innate immunity^35^. While the FXR/RXR pathway was ranked among the top five canonical pathways in seven spheroid types in our study, it lacked a directional Z-score in the IPA results. However, the consistent upregulation of its associated proteins raises the possibility that the pathway may be activated in response to DPM.

Since LXR/RXR and FXR/RXR are major transcriptional factors involved in lipid and glucose metabolism, perturbations in one pathway may provoke a complementary response from the other^34^. In addition, key players of these pathways are known to be affected by PM^1,2^. For example, the role of LXR receptor in macrophage function and foam cell transformation, which contributes to PM-induced atherosclerosis, has been well documented^36^. Chronic exposure to traffic-related PM has also been shown to induce lung injury and LXR/RXR activation in a rat model^29^. FXR signaling pathway can be activated by organic components in PM, thereby disrupting bile acid synthesis and lipid metabolism^37^. In this regard, we hypothesized that the LXR or FXR agonists/antagonists could be used to actively regulate the detrimental effects induced by DPM, assuming the proteins identified in our proteomic analysis, which are associated with these pathways, are indeed regulated by LXR or FXR. From this perspective, we decided to explore several downstream proteins in these pathways and examined how protein expression levels were affected by DPM treatment.

### Western blot analysis

Among the proteins that exhibited statistically significant increases (> 1.5-fold) in spheroids exposed to the highest DPM concentrations compared to unexposed spheroids at 48 h (Table S20), APOA1 and APOH were further validated by Western blot analysis. Both APOA1 and APOH are involved in lipid transport and metabolism and are linked to key signaling pathways, including LXR/RXR activation and FXR/RXR activation, which were commonly observed across all spheroid types. KNG1 was excluded from the Western blot analysis because its function is primarily related to blood coagulation as a protease inhibitor, rather than lipid transport and metabolism. Western blot analysis was conducted on eight types of spheroids exposed to increasing DPM concentrations (Fig. S7), revealing a strong correlation between the expression levels of these proteins (APOA1 and APOH) and DPM concentrations as determined by the quantitative MS data.

APOA1 functions as the primary transporter of plasma HDLs while apolipoprotein B-100 (APOB) serve as the primary transporter of low-density lipoproteins (LDLs). The ratio of APOA1 and APOB is often used as a risk factor for various metabolic diseases. Due to its clinic-pathological characteristics, several studies have reported the effects of PM exposure on blood lipoproteins including APOA1 and APOB, and their relevance to the metabolic diseases^38–40^. For example, higher ambient PM levels were associated with significant reductions in APOA1 levels^39^. On the other hand, APOA1 significantly decreased after short term exposure (0–6 h) of PM, but the percent changes in APOA1 levels after long term exposure (7 h to 5 d) were not statistically significant when considering factors such as age, gender, body mass, exercise, time trend, and temperature^38^. In contrast, our proteomic data showed that APOA1 levels increased with DPM treatment across all eight spheroid types. Although it is not clear why these results are inconsistent, the exposure time, age of the participants, PM sources, and constituents may be considered to explain these discrepancies. On the other hand, the association of APOH with PM has not been reported yet. APOH binds to LDL and its plasma level is associated with the oxidative susceptibility of LDL in human, suggesting its role of inhibition of LDL oxidation^41^.

In addition to their canonical functions, the beneficial roles of APOH and APOA1 in inflammation have been documented. APOA1 can bind to LPS and lipoteichoic acid, preventing these endotoxins from binding to toll-like receptor 4 and thereby reducing pro-inflammatory signaling pathways that induce sepsis pathology^42,43^. APOH has been suggested as a biomarker for sepsis^44^ systemic lupus erythematosus^45^ and it can also bind to LPS, regulating its uptake^46^. All of these functions contribute to the reduction of inflammation. Therefore, it is likely that spheroids exposed to DPM were actively adjusted to prevent inflammation through regulating expression of these proteins.

### Possible mechanism for APOA1 increase

**Fig. 4:**
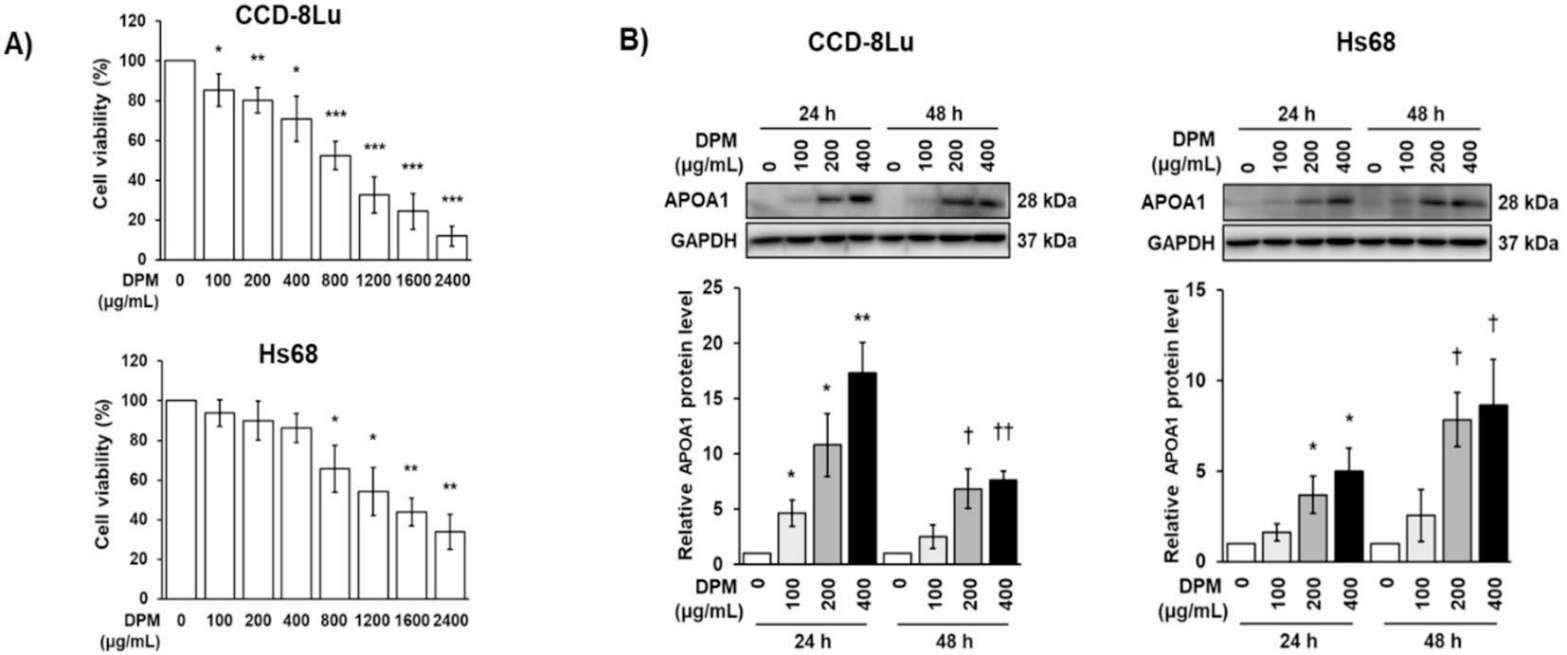
APOA1 was upregulated by DPM treatment in CCD-8Lu and Hs68 cells. CCD-8Lu and Hs68 cells were plated at a density of 4,000 cells/cm² in a 60 mm dish and incubated with various concentrations of DPM for 24–48 h at 70% confluence. (**A**) Cell viability determined by MTS assay after 24 h (**B**) The protein expression levels of APOA1 was determined by Western blot analysis. Each value is the mean ± SEM of three independent experiments. * *p* < 0.05, ** *p* < 0.01, and *** *p* < 0.001 vs. 24 h control, † *p* < 0.05 and †† *p* < 0.01 vs. 48 h control. Unlabeled values were not statistically significant.

Among the five proteins that showed statistically significant increases (*P*-value < 0.05, > 1.5-fold) in spheroids exposed to the highest DPM concentrations compared to unexposed spheroids at 48 h across all eight spheroid types, we focused on APOA1, which exhibited consistent increases with rising DPM concentrations across each spheroid type. For validation, we chose two different fibroblast cell lines, CCD-8Lu (human lung fibroblasts) and Hs68 (human dermal fibroblasts) cells as model systems. To examine that these cell lines can be used as representative for 3D spheroids derived from primary cells, we first determined the expression levels of proteins (APOA1, and APOH) in CCD-8Lu 3D spheroids. The changes in expression of these proteins in CCD-8Lu were consistent with those observed in 3D spheroids of primary lung fibroblasts, LF-S (Fig. S8A). Furthermore, CCD-8Lu and Hs68 cells cultured on a 2D plate showed similar expression patterns for these proteins compared to those cultured in 3D spheroids (Fig. S8B). Therefore, we used the 2D cultures of CCD-8Lu and Hs68 cells for the rest of our mechanism study for validation. Next, the optimal concentrations of DPM for CCD-8Lu and Hs68 cells were determined based on their cell viability (Fig. 4A). CCD-8Lu and Hs68 cells showed approximately 50% viability with DPM treatments of 800 µg/mL and 1400 µg/mL, respectively. Both cell lines maintained over 70% viability up to a DPM concentration of 400 µg/mL. The expression of APOA1 was positively correlated with the DPM concentration up to 400 µg/mL at both 24 and 48 h of incubation in both cell types. (Fig. 4B).

**Fig. 5:**
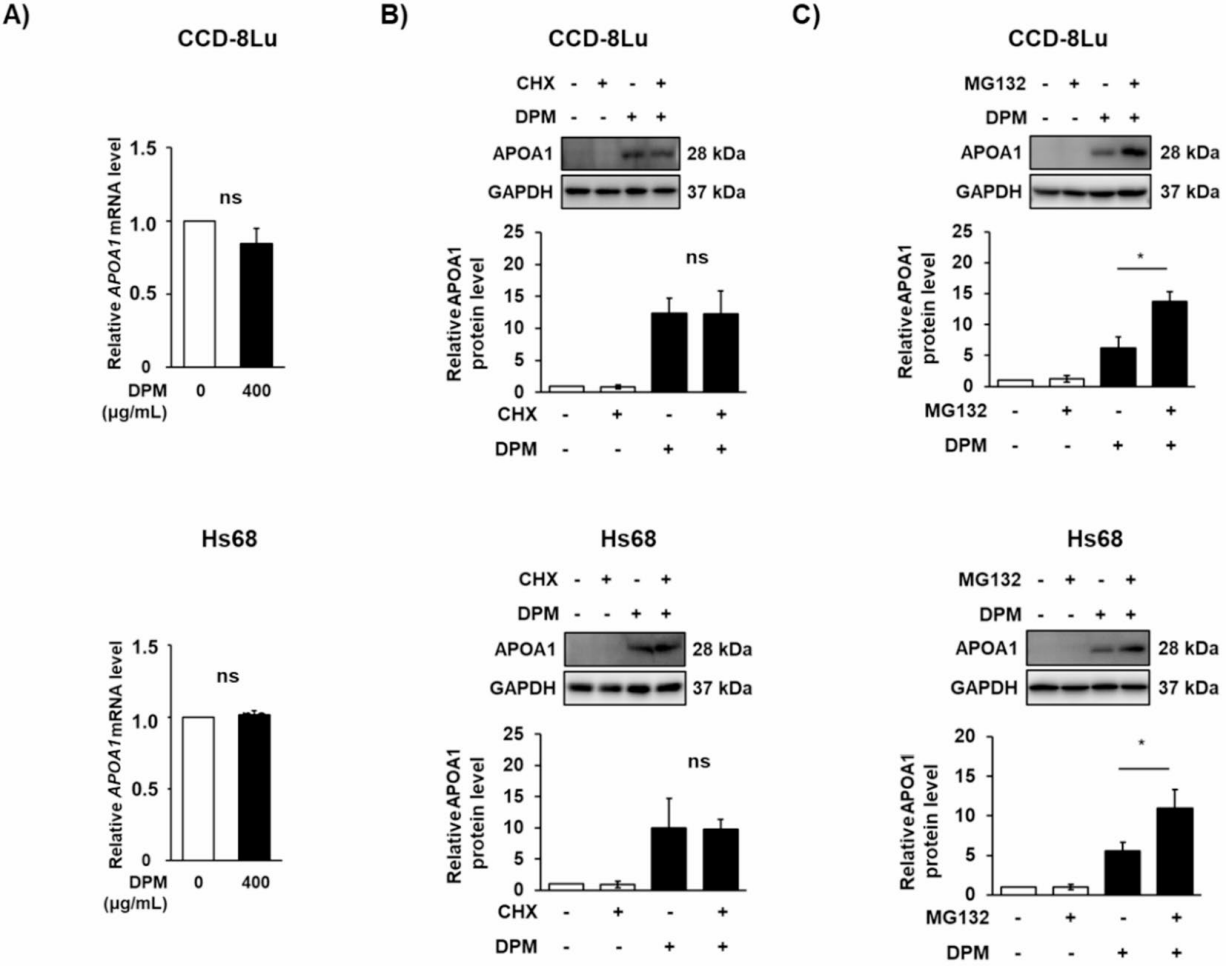
The effect of DPM on protein degradation of APOA1. CCD-8Lu and Hs68 cells were plated at a density of 4,000 cells/cm² in a 60 mm dish and incubated with or without 400 µg/mL DPM for 24 h at 70% confluence. (**A**) The mRNA level of APOA1 was determined by RT-qPCR analysis. (**B**) Western blot analysis of APOA1. CCD-8Lu and Hs68 cells were incubated with or without 400 µg/mL DPM. 100 µg/mL CHX was added after 12 h of DPM treatment, and then treated cells were harvested simultaneously at 24 h of DPM treatment. (**C**) Western blot analysis of APOA1. CCD-8Lu and Hs68 cells were incubated with or without 400 µg/mL DPM. 20 µM MG132 was treated for 6 h before cells were harvested at 24 h of DPM treatment. Each value is the mean ± SEM of three independent experiments. * p < 0.05. ns, not significant.

The expression level of APOA1 can be actively regulated at three different levels: transcription, translation and degradation. To elucidate the underlying mechanisms, we first investigated whether the APOA1 mRNA level was affected by DPM treatment. Figure 5A shows that the APOA1 mRNA level was maintained regardless of DPM treatment in both CCD-Lu and Hs68 cells. Consistent with these data, treatment with the PPARα antagonist GW6471, LXR antagonist GSK2033, and the LXR agonist T0901317, all of which are well-known transcriptional regulators for APOA1, did not affect the APOA1 mRNA level (Fig. S9A and S9B). Furthermore, the expression levels of LXRβ slightly decreased upon DPM treatment (Fig. S9C). These data suggest that the enhanced expression of APOA1 in response to DPM treatment was not due to increased transcription. In addition, our proteomic data revealed that most ribosomal proteins involved in translation (protein synthesis) were found to be decreased by DPM treatment across all eight spheroid types (Table S20). In this regard, it is unlikely that protein translation of APOA1 was enhanced by DPM treatment.

Next, we explored how DPM treatment affected the degradation of APOA1. To examine the degradation of APOA1, we treated CCD-8Lu and Hs68 with CHX, a protein synthesis inhibitor. APOA1 was barely detected in both CCD-8Lu and Hs68 that were not treated with DPM regardless of CHX treatment (Fig. 5B). With DPM treatment, APOA1 levels increased and remained unchanged in the presence of CHX (Fig. 5B), suggesting that degradation of APOA1 was barely observed in DPM-treated cells. Moreover, proteasome-dependent degradation inhibitor MG132 further increased APOA1 expression in the DPM-treated cells (Fig. 5C). To reveal the proteins involved in the degradation pathway of APOA1, we focused on tripartite motif (TRIM) family of proteins, which function as E3 ligases for ubiquitin-dependent proteasome degradation. TRIM proteins are classified into nine families based on their domain structure and play important roles in a broad range of innate immunity processes^47^. Among the various TRIM isoforms, tripartite motif-containing protein 15 (TRIM15) has been reported to be involved in regulation of APOA1 in pancreatic cancer cells^48^. In our MS data, TRIM15 (Q9C019) was identified and had quantifiable values in six spheroid types (NEC-S, B/TEC-S, LF-S, SAEC-S, M-S, and CAEC-S); however, the protein did not show statistical significance across various concentrations of DPM at both 24 and 48 h in the two-way ANOVA analysis. Alternatively, we examined the protein expression levels of the TRIM C-IV family, which belongs to the same TRIM family as TRIM15, in the quantitative MS data (Table S11 ~ S18). Table S22 summarizes the fold change ratios of TRIM C-IV family proteins, which exhibited statistical significance (*P*-value < 0.05) depending on DPM concentrations from the two-way ANOVA, comparing unexposed spheroids to those exposed to each DPM concentration. Upon DPM treatment, TRIM26 was noticeably decreased in LF-S, while TRIM38 marginally decreased (Table S22). On the other hand, TRIM25 and TRIM26 significantly decreased in DF-S (Table S22). However, protein expression of TRIM26 in both CCD-8Lu and Hs68 was barely detected in Western blot analysis (data not shown). Therefore, we chose TRIM25 as possible E3 ligases for APOA1 in Hs68 cells.

**Fig. 6:**
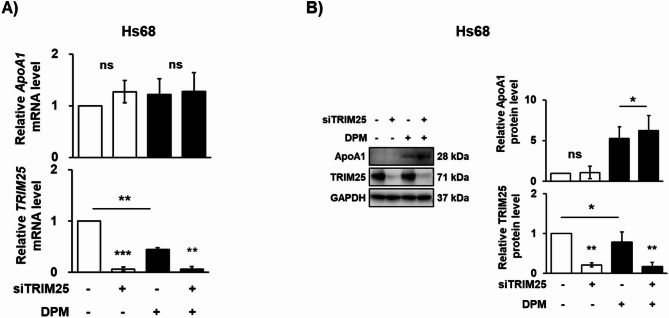
APOA1 was regulated by TRIM dependent degradation in Hs68 cells. Hs68 cells were plated at a density of 4,000 cells/cm² in a 60 mm dish and transfected with 50 nM TRIM25 siRNA for 48 h at 70% confluence, respectively, and then incubated with 400 µg/mL DPM for 24 h. (**A**) The mRNA levels of APOA1, TRIM25 and GAPDH were determined by RT-qPCR. (**B**) The protein levels of APOA1 and TRIM25 were evaluated by western blot analysis. Each value is the mean ± SEM of three independent experiments. * p < 0.05, ** p < 0.01, and *** p < 0.001.

To explore how these TRIM proteins affect APOA1 level, we constructed siRNAs and treated Hs68 cells in the presence or absence of DPM. RT-qPCR analysis showed that the mRNA expression levels of TRIM25 in Hs68 cells decreased upon appropriate siRNA treatments (Fig. 6A). Interestingly, DPM treatment also led to a reduction in the mRNA levels of TRIM25, suggesting that DPM downregulates the transcription of this protein (Fig. 6A). APOA1 mRNA was maintained regardless of siRNA treatments. However, knockdown of TRIM25 protein by siRNA treatment further enhanced APOA1 protein expression levels in DPM-treated cells (lane 4 in Fig. 6B). Therefore, we concluded that impaired degradation of APOA1 via TRIM25 is responsible for the increased levels of APOA1 under DPM treatment in Hs68 cells. Notably, APOA1 was not detected even when TRIM25 was depleted by siRNAs (lane 2 in Fig. 6B) in the absence of DPM treatment. These data suggest that a degradation pathway other than TRIM-dependent degradation should be involved in APOA1 degradation under normal conditions. In fact, a recent study by Georgila et al.. demonstrated that post-transcriptional regulation through autophagy-dependent degradation of APOA1 plays an important role in HepG2 hepatoma cells^49^. The half-life of APOA1 in HepG2 cells was approximately 30 min, implying that APOA1 is rapidly degraded by autophagy and thus active protein synthesis is required in order to maintain intracellular APOA1 level under normal conditions. Therefore, it is likely that APOA1 is actively degraded by autophagy in CCD-8Lu and Hs68 cells under normal conditions.

To explore the possible accumulation of autophagosomes under DPM treatment, we measured the protein expression levels of autophagosome components, LC3B-II complex and p62, in response to increasing DPM concentrations. The autophagosome components of LC3B-II and p62, along with APOA1 were accumulated as DPM concentrations increased to 200 µg/mL in CCD-8Lu cells (Fig. S10). Mild concentrations of DPM treatment (< 400 µg/mL) might contribute to the stabilization of APOA1 through autophagosome accumulation. However, 400 µg/mL of DPM treatment reverted the protein expression levels of both LC3B-II complex and p62 to normal conditions. Therefore, APOA1 stabilization through autophagosome accumulation appeared to be impaired at 400 µg/mL of DPM treatment. In this condition, TRIM-dependent proteasome degradation is more involved in maintaining APOA1 stability because the APOA1 level further increased upon depletion of TRIM isoforms.

In contrast to our original hypothesis, we found that the enhanced APOA1 expression by DPM treatment is not caused by transcriptional factors such as PPARα and LXR, but rather by impaired autophagy and proteasome-dependent degradation. Considering that autophagy is activated and LC3B-II complex increases by DPM treatment^50,51^ and that other toxic materials can be eliminated through autophagy, an increased autophagy flux can improve stress resistance and reduce apoptosis^52^. In our case, it is likely that autophagosomes were accumulated but not degraded, leading to the stabilization of APOA1 at certain DPM concentrations (< 400 µg/mL). At higher concentrations, autophagosomes decreased and autophagy was activated, such that both LC3-II and p62 levels decreased, suggesting that the regulation of APOA1 occurs by proteasome-dependent degradation. While a previous study by Georgila et al.. demonstrated that APOA1 undergoes rapid autophagy-dependent degradation in HepG2 hepatoma cells under basal conditions^49^ the impact of environmental toxicants on this degradation pathway has not been previously reported. To our knowledge, this is the first study to show that DPM exposure increases APOA1 levels by disrupting both autophagy- and proteasome-mediated degradation. These findings reveal a previously unrecognized mechanism of environmental regulation that modulates APOA1 abundance independently of transcriptional activation. Since recent studies emphasize the role of APOA1 in reducing inflammation, further studies to reveal how APOA1 stabilization contributes to mitigating the deleterious effects of DPM may be useful in elucidating the role of APOA1 as a target for combating DPM-induced toxicity.

## Supplementary Information

Below is the link to the electronic supplementary material.

Supplementary Material 1

Supplementary Material 2

## Data Availability

The data analyzed in the current study are available from the corresponding author on reasonable request. The mass spectrometry data have been deposited to the ProteomeXchange Consortium via the PRIDE^53^ partner repository with the dataset identifier PXD056979 (https://www.ebi.ac.uk/pride/archive/projects/PXD056979).
