# Supplementary material for: Diesel particulate matter-induced proteomic changes in three-dimensional spheroids derived from human primary cells of various tissue origins
Source: Sci Rep. 2025 Sep 24;15:32734. doi: 10.1038/s41598-025-17873-7 (PMC12460833; doi:10.1038/s41598-025-17873-7)
Supplement: Supplementary file 2 — Supplementary Material 2 [file 41598_2025_17873_MOESM2_ESM.docx]

**Diesel particulate matter-induced proteomic changes in three-dimensional spheroids derived from human primary cells of various tissue origins**

Yoon Jin Cho^1,2†^, Hae Dong Jeong^1†^, Young June Jeon^1,3^, Soobin Choi^1^, Ji Hyun Back^1^,

Ji Hun Wi^1, 4^, Yae Eun Park^1^, Seung-Hee Gwak^1^, Mi Jung Ji^5^, Hyun-Mee Park^5^, Hyuk Jeong^2^, So Yeon Kim^1, 6, 7*^, and Ji Eun Lee^1*^

^1^Chemical and Biological Integrative Research Center, Biomedical Research Division, Korea Institute of Science and Technology, Seoul, 02792, Republic of Korea

^2^Department of Chemistry, Sookmyung Women's University, Seoul, 04310, Republic of Korea

^3^﻿Department of Bioengineering, Hanyang University, Seoul, 04763, Republic of Korea

^4^Department of Biotechnology, College of Life Sciences and Biotechnology, Korea University, Seoul, 02841, Republic of Korea

^5^ Advanced Analysis and Data Center, Research Resources Division, Korea Institute of Science and Technology, Seoul, 02792, Republic of Korea

^6^ Division of Bio-Medical Science and Technology, KIST School, Korea University of Science and Technology (UST), Seoul, 02792, Republic of Korea

^7^ KHU-KIST Department of Converging Science and Technology, Kyung Hee University, Seoul, 02447, Republic of Korea

^†^These two authors equally contributed to this study.

^*^Correspondence to:

So Yeon Kim (e-mail: soyeonkim@kist.re.kr; telephone 82-2-958-5914; fax 82-2-958-5909), 5, Hwarang-ro 14-gil Seongbuk-gu Seoul, 02792, Republic of Korea

Ji Eun Lee (e-mail: jelee9137@kist.re.kr; telephone 82-2-958-6422; fax 82-2-958-5909), 5, Hwarang-ro 14-gil Seongbuk-gu Seoul, 02792, Republic of Korea
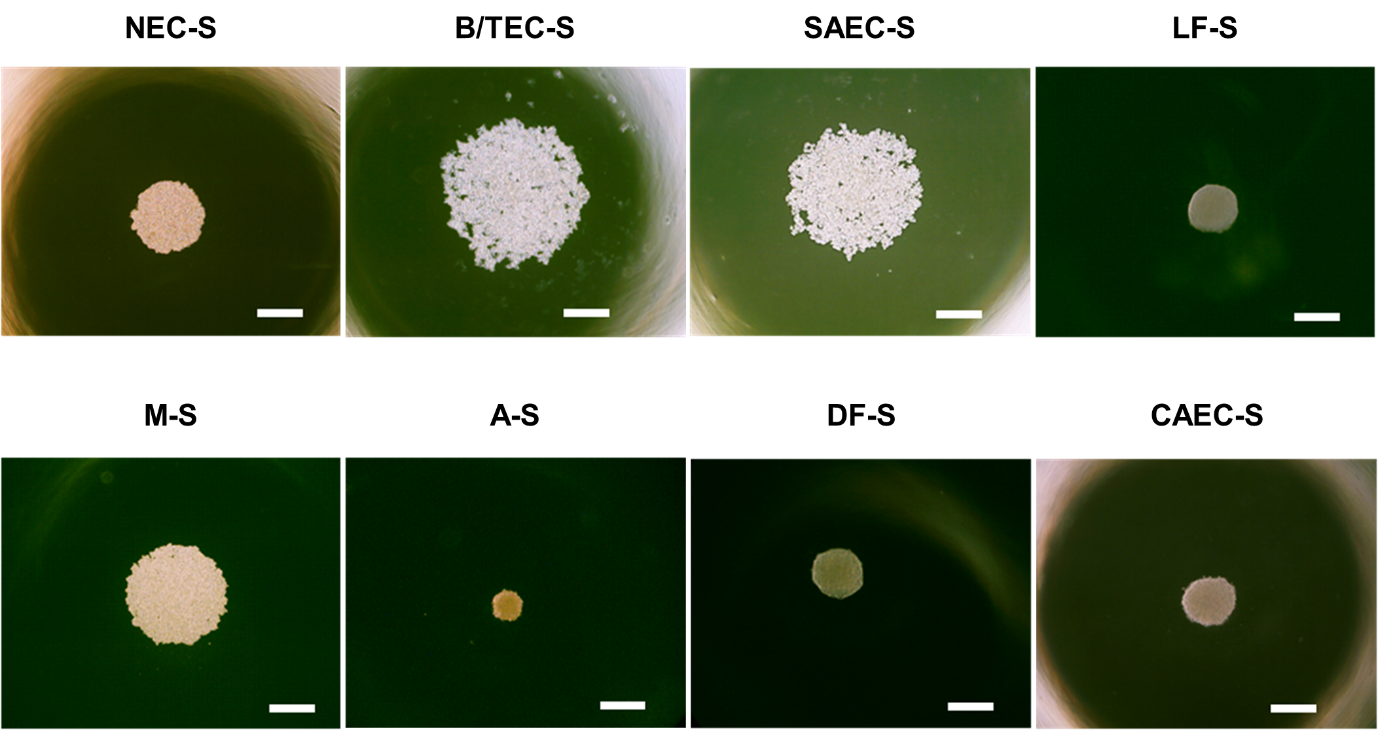


**Supplementary Figure 1**. Optical images of spheroids formed from eight types of human primary cells one day after seeding by phase contrast microscopy (scale bar, 500 μm). Human nasal epithelial cells (NECs, 4.0 × 10^3^ cells), bronchial/tracheal epithelial cells (B/TECs, 6.0 × 10^3^ cells), small airway epithelial cells (SAECs, 6.0 × 10^3^ cells), lung fibroblasts (LFs, 9.0 × 10^3^ cells), microglia (M, 8.0 × 10^3^ cells), astrocytes (As, 5.0 × 10^3^ cells), dermal fibroblasts (DFs, 6.0 × 10^3^ cells), and coronary artery endothelial cells (CAECs, 1.2 × 10^4^ cells), were, respectively, seeded with 200 μL of the corresponding cell culture medium in ultra-low attachment 96 well plates, and then the spheroids from the eight types of human primary cells were observed one day after seeding. The abbreviations for each spheroid type are as follows: NEC-S for nasal epithelial cell-derived spheroids, B/TEC-S for bronchial/tracheal epithelial cell-derived spheroids, SAEC-S for small airway epithelial cell-derived spheroids, LF-S for lung fibroblast-derived spheroids, DF-S for dermal fibroblast-derived spheroids, CAEC-S for coronary artery endothelial cell-derived spheroids, M-S for microglia-derived spheroids, and A-S for astrocyte-derived spheroids.


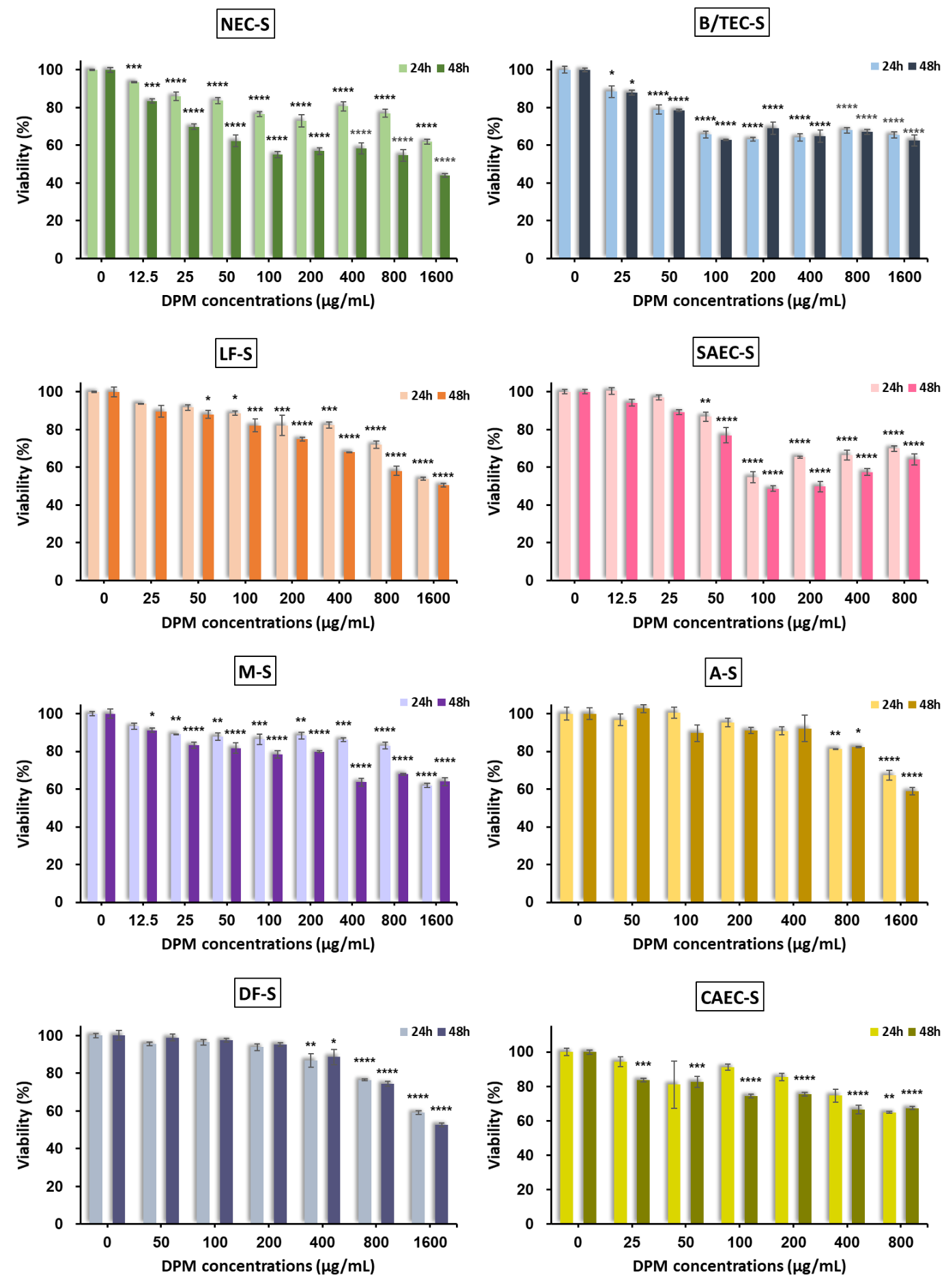


**Supplementary Figure 2**. Viability of spheroids derived from eight primary cell types exposed to varying concentrations of diesel particulate matter (DPM) solutions. Data are expressed as mean ± SEM (n=3). Statistical significance was evaluated using one-way ANOVA for each time point, followed by Tukey's multiple comparison tests. Asterisks above each bar indicate a significant difference between each concentration and the control group (* p<0.05, ** p<0.01, *** p<0.001, **** p<0.0001).


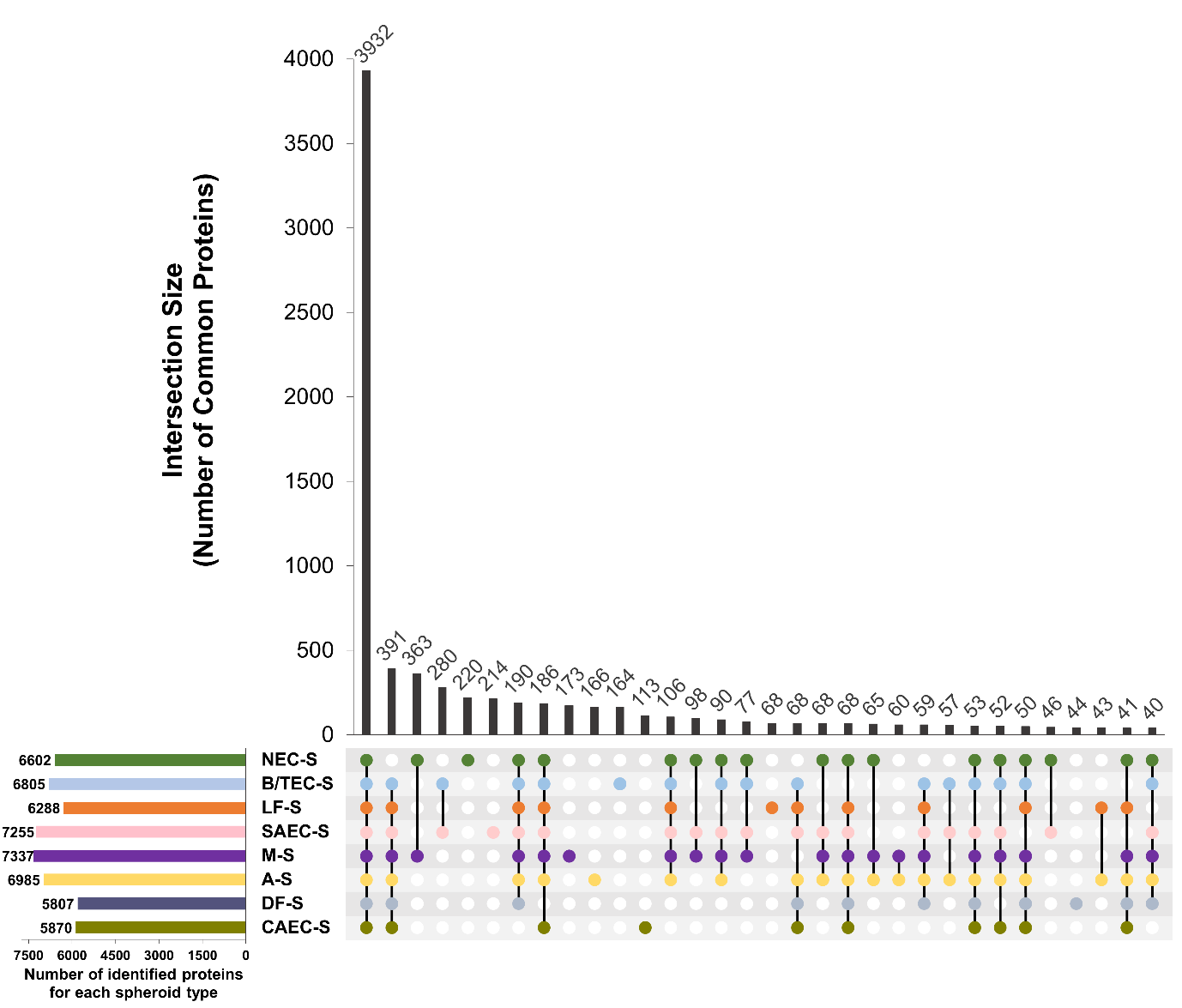


**Supplementary Figure 3**. UpSet plot depicting the common proteins identified among the eight spheroid types. The plot displays intersections of protein identifications with intersection sizes greater than or equal to 40 proteins. The plot displays intersections of protein identifications with intersection sizes greater than or equal to 40 proteins, highlighting the shared and unique protein profiles among the different spheroid types.


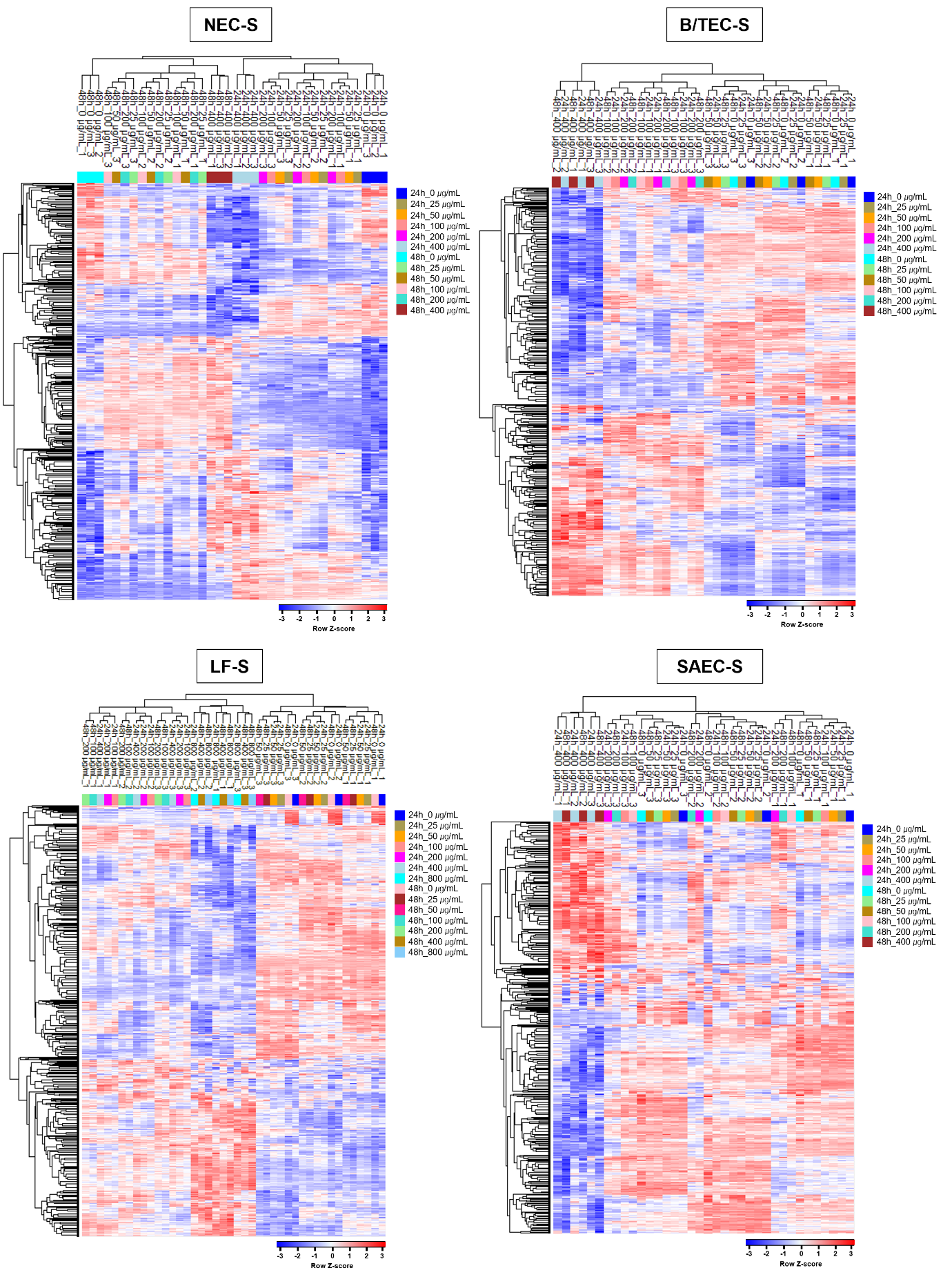


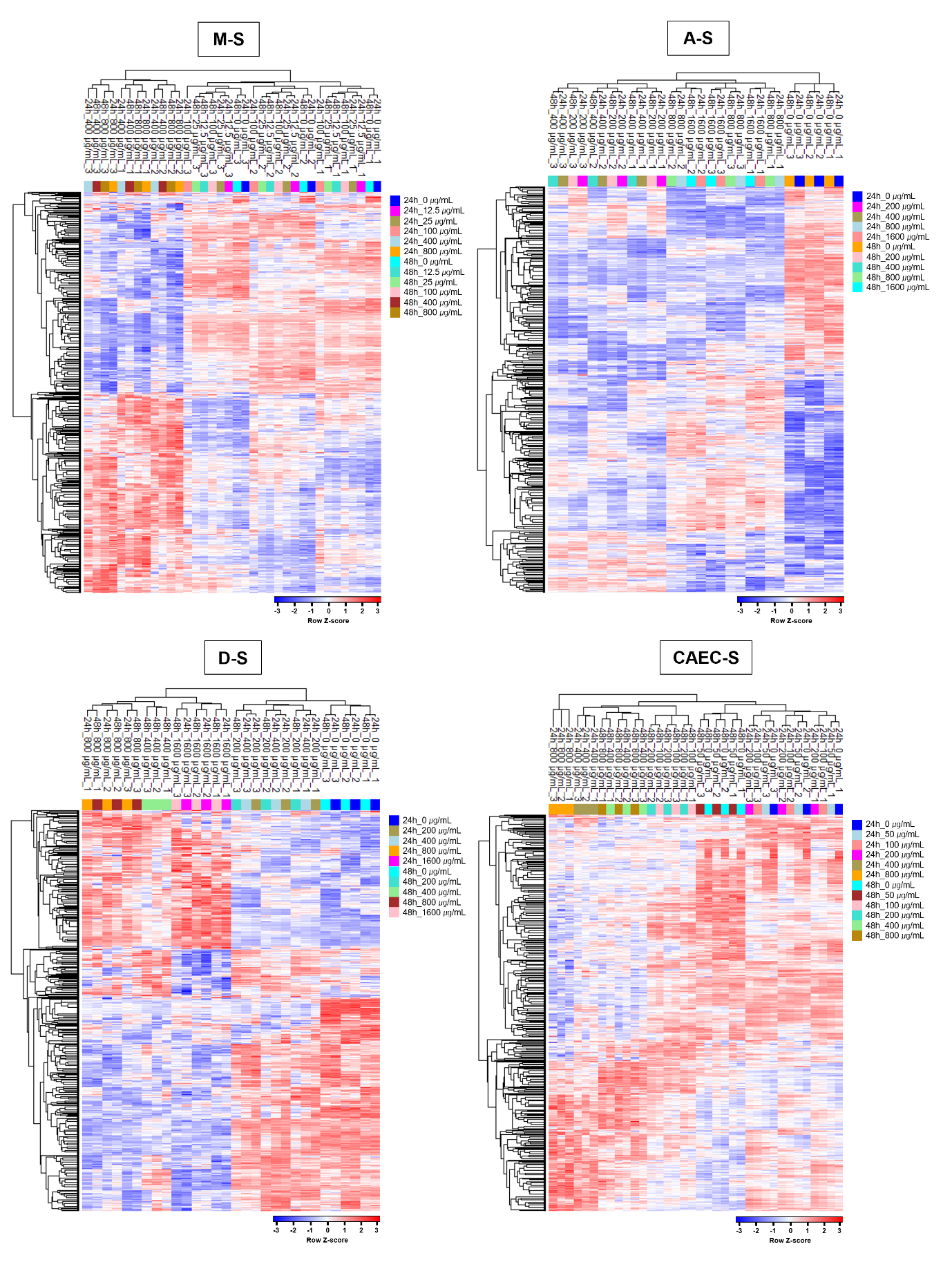


**Supplementary Figure 4**. Heat maps displaying hierarchical clustering of the proteins with statistical significance (*P*-value <0.05) based on DPM concentrations from two-way ANOVA analysis for eight spheroid types. Rows represent individual proteins while columns correspond to three technical replicates of LC-MS/MS runs for each DPM concentration. Colored bars below the sample dendrogram represent the exposure condition of each sample, specified by the treatment time and DPM concentration. Hierarchical clustering of the proteins was performed on log_2_-transformed normalized abundance values after Z-score normalization of the data using Perseus software (1.6.14.0).


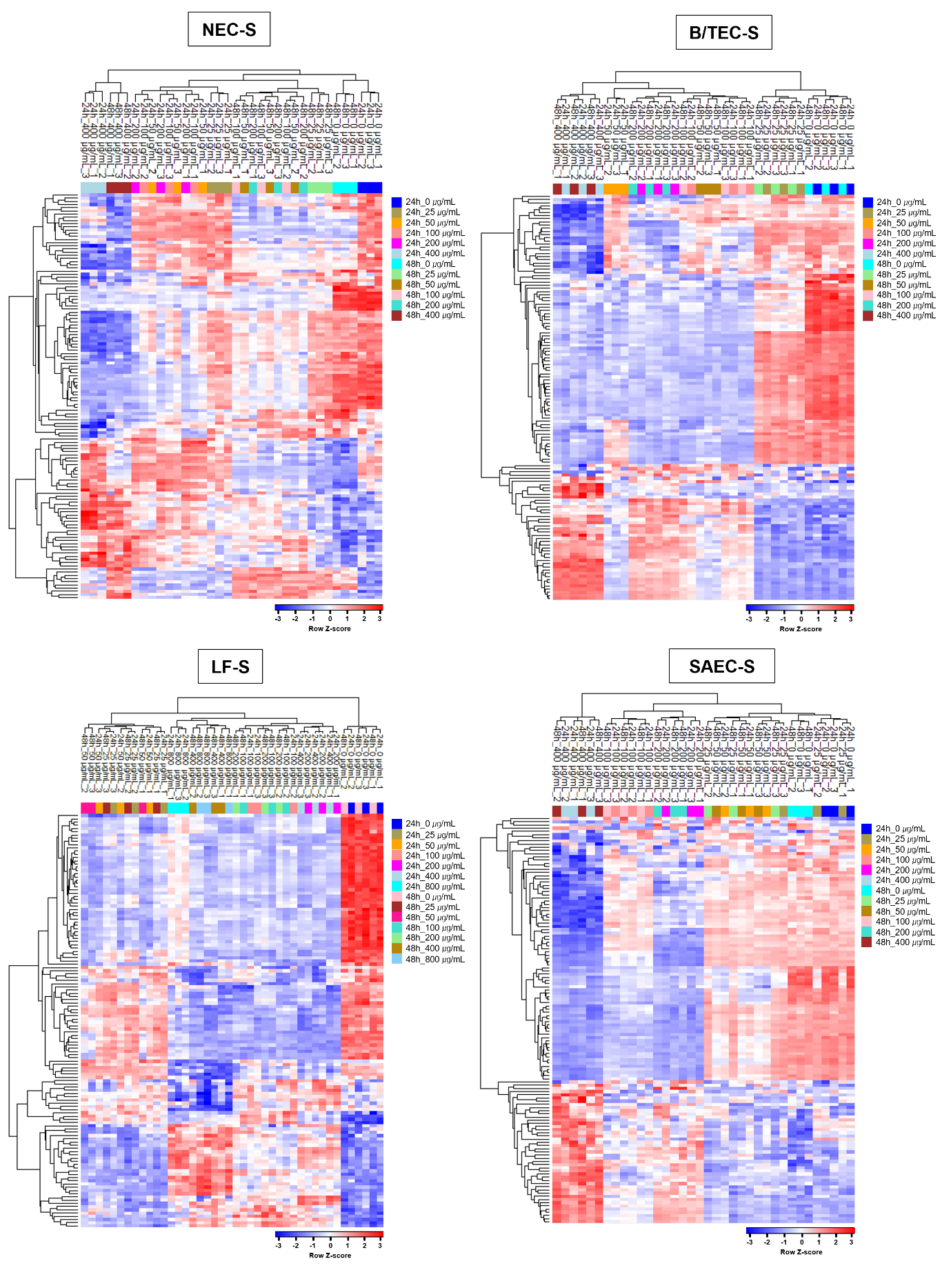

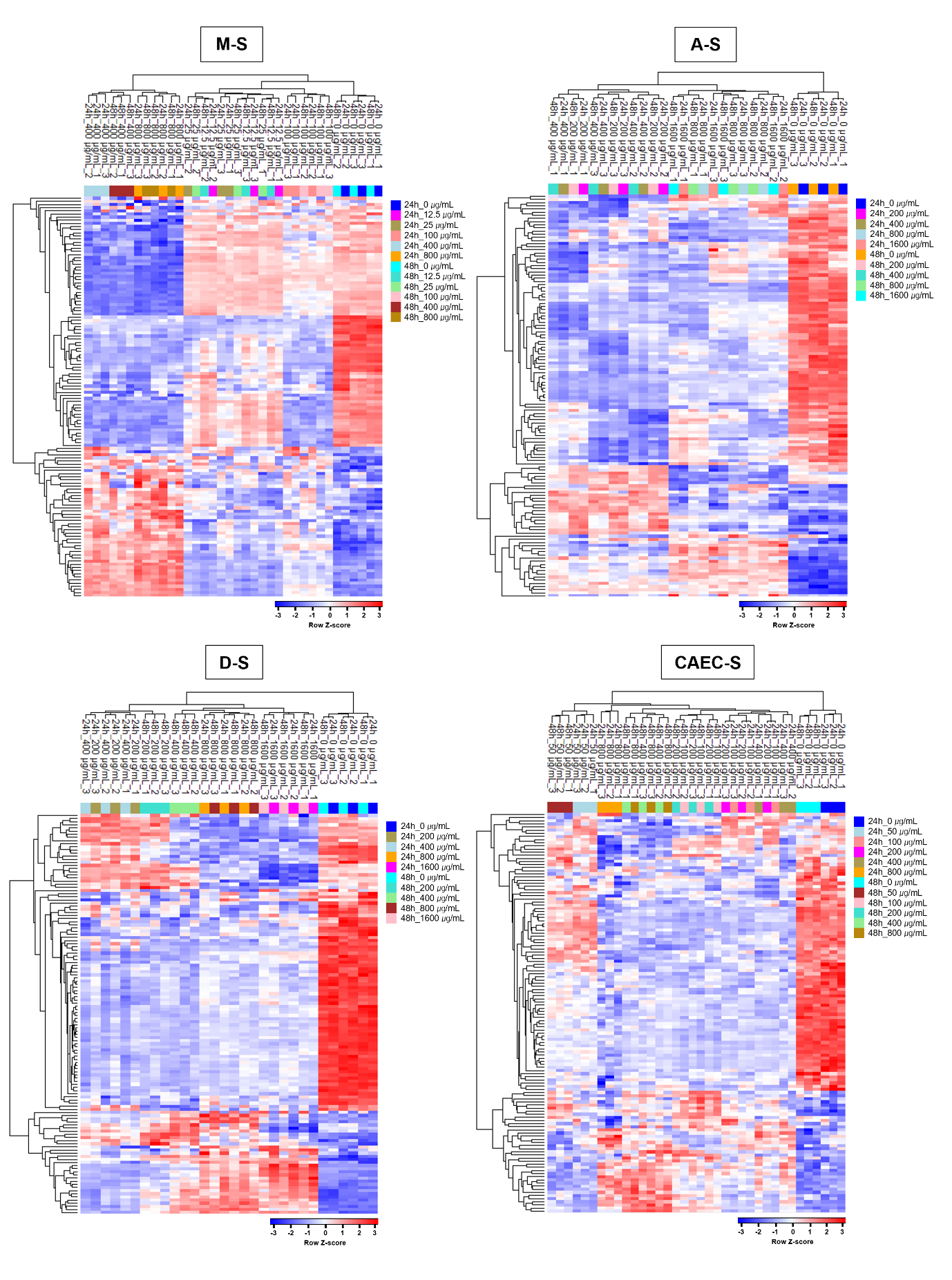


**Supplementary Figure 5**. Heat map exhibiting hierarchical clustering of 128 proteins showing statistical significance (P-value <0.05) across eight spheroid types based on DPM concentrations from two-way ANOVA analysis. Rows represent individual proteins while columns correspond to three technical replicates of LC-MS/MS runs for each of the DPM concentrations. Colored bars below the sample dendrogram represent the exposure condition of each sample, specified by the treatment time and DPM concentration. Hierarchical clustering of the 128 proteins was performed on log-transformed normalized abundance values after Z-score normalization of the data using Perseus software (1.6.14.0).


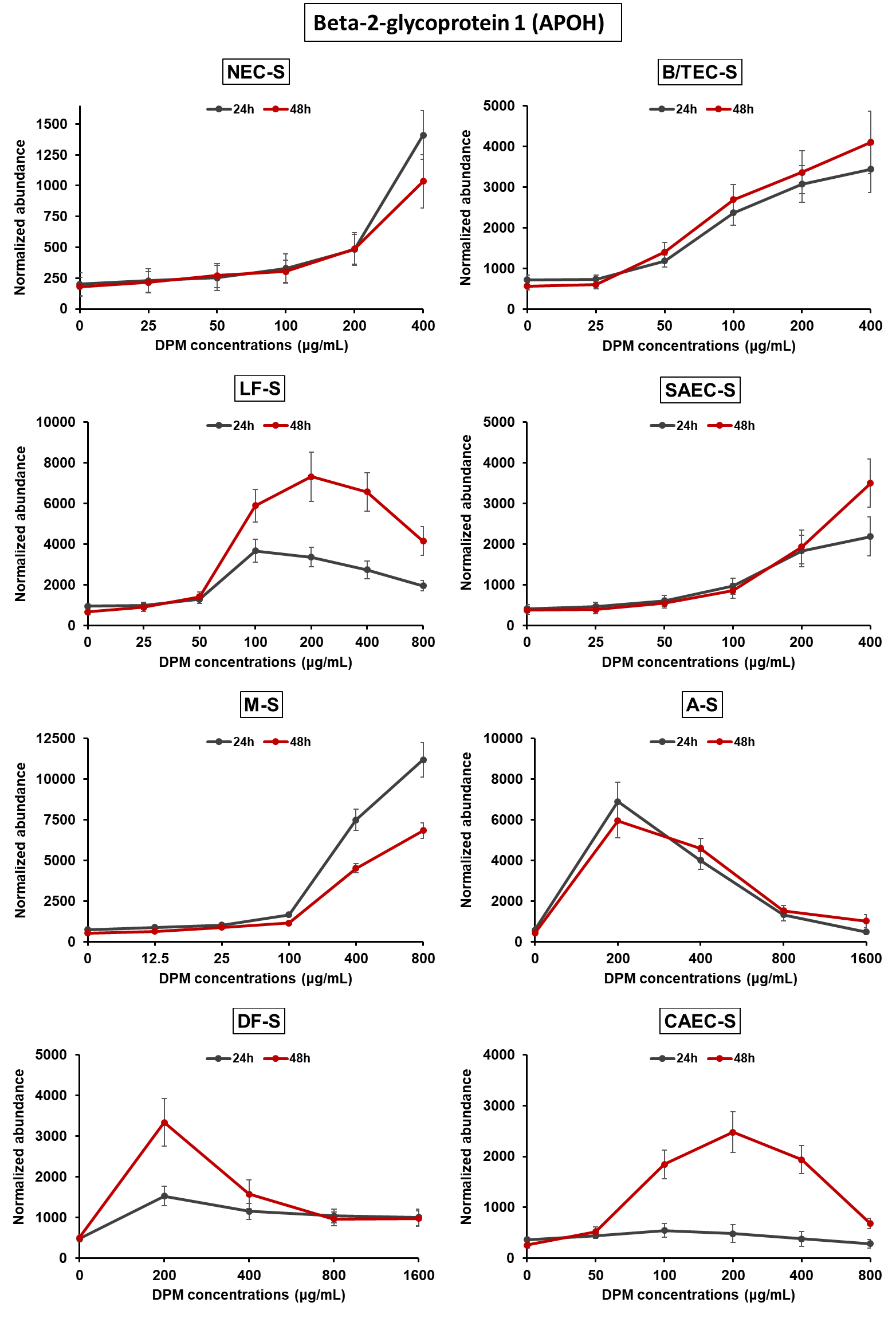


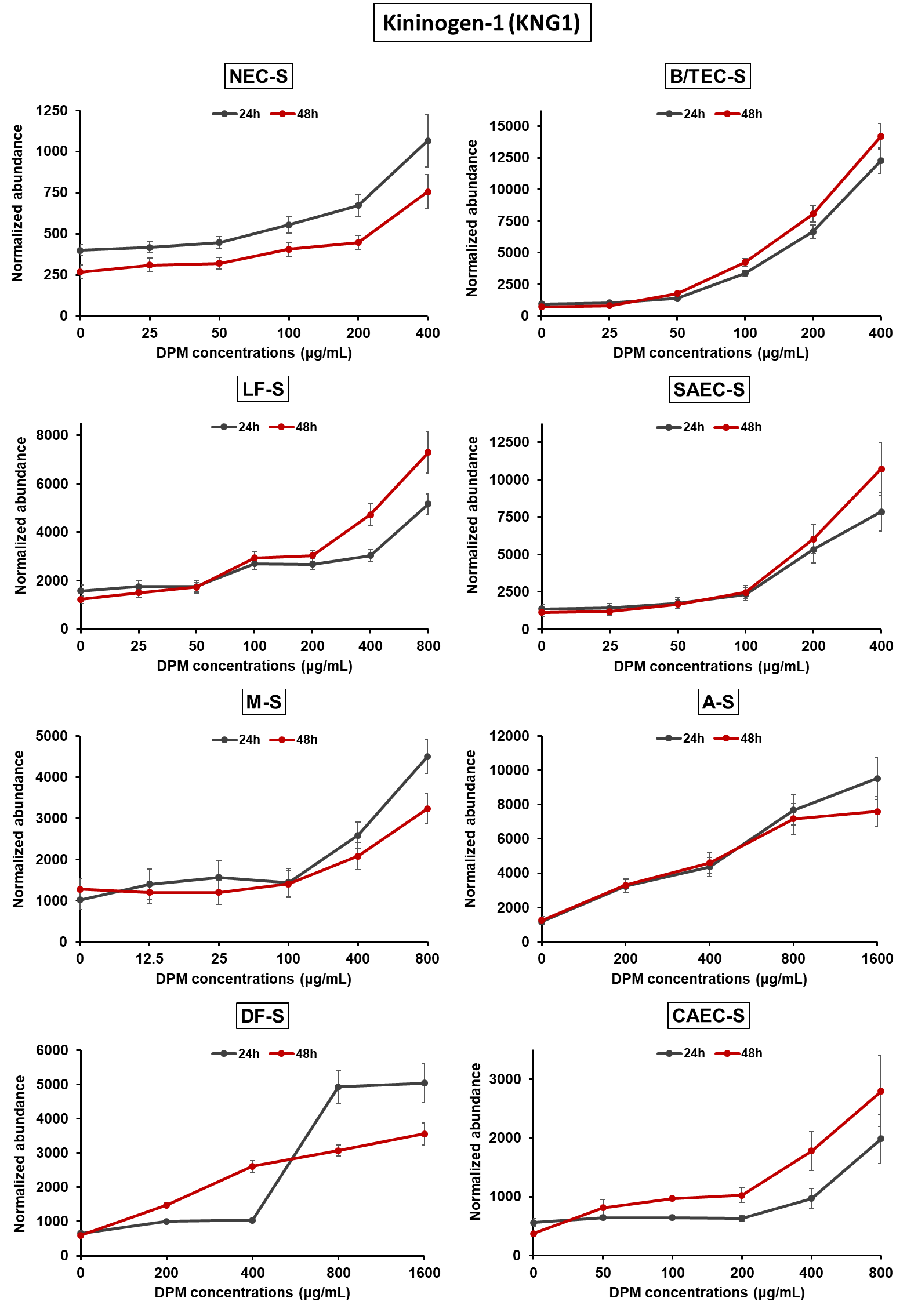


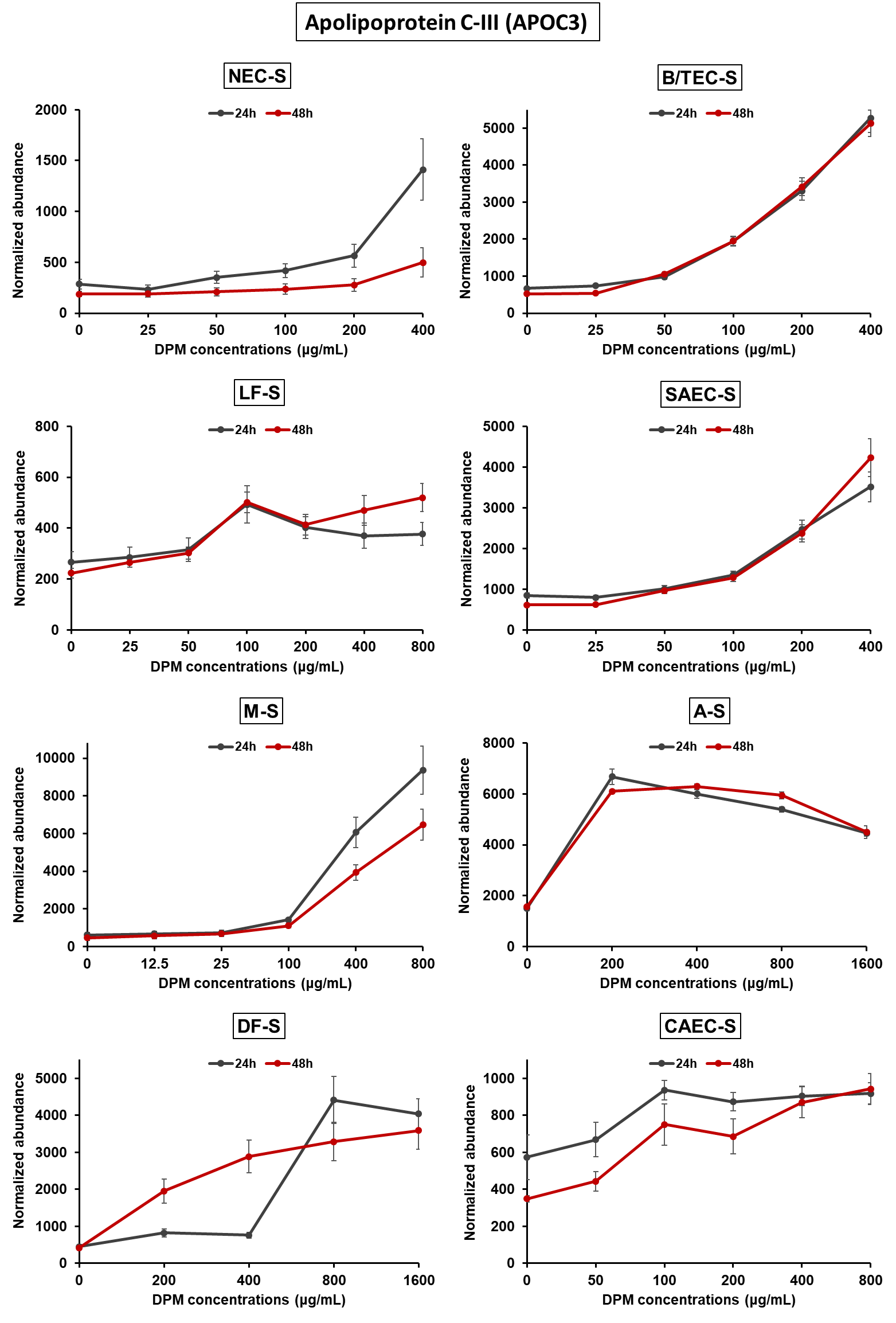


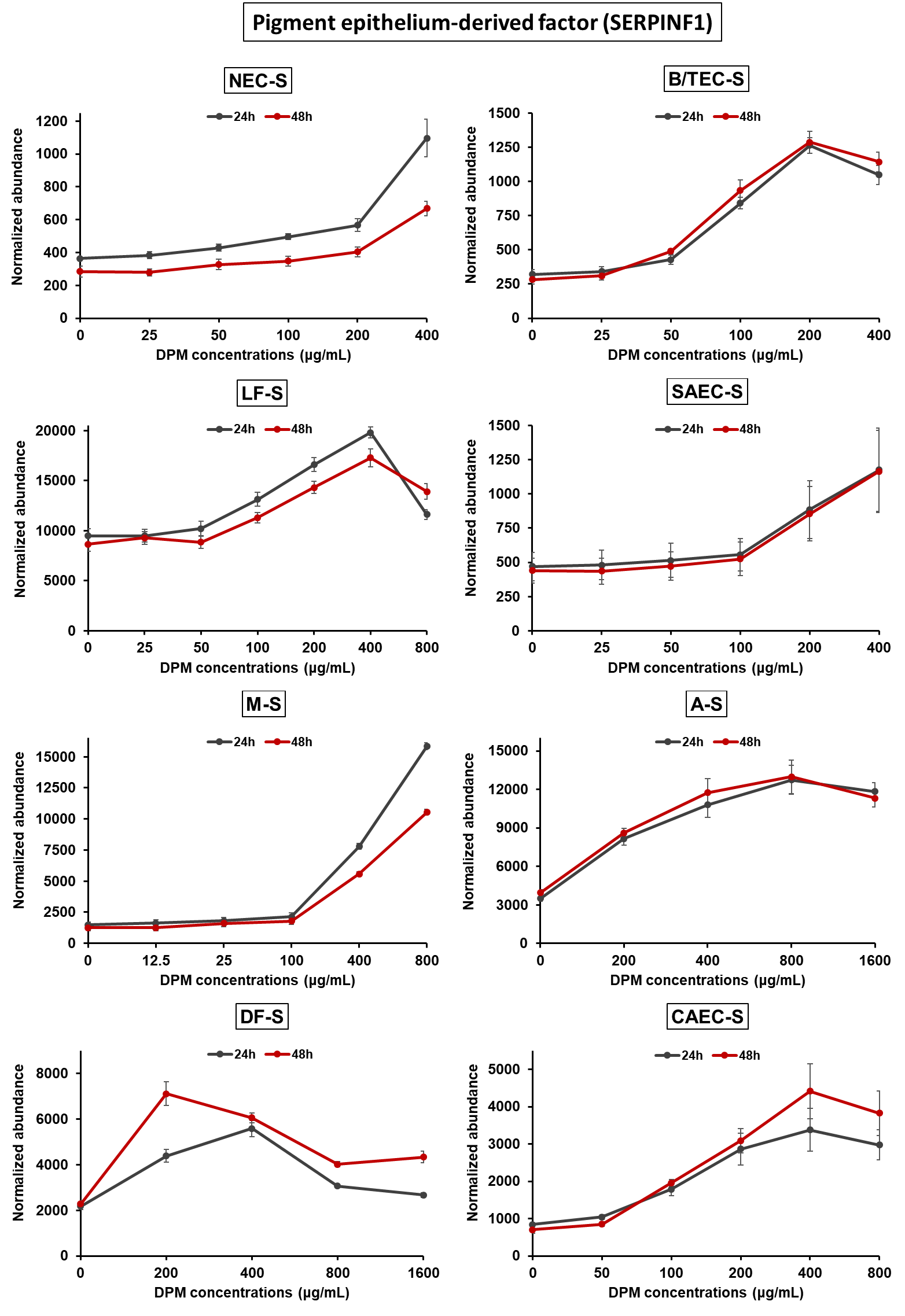


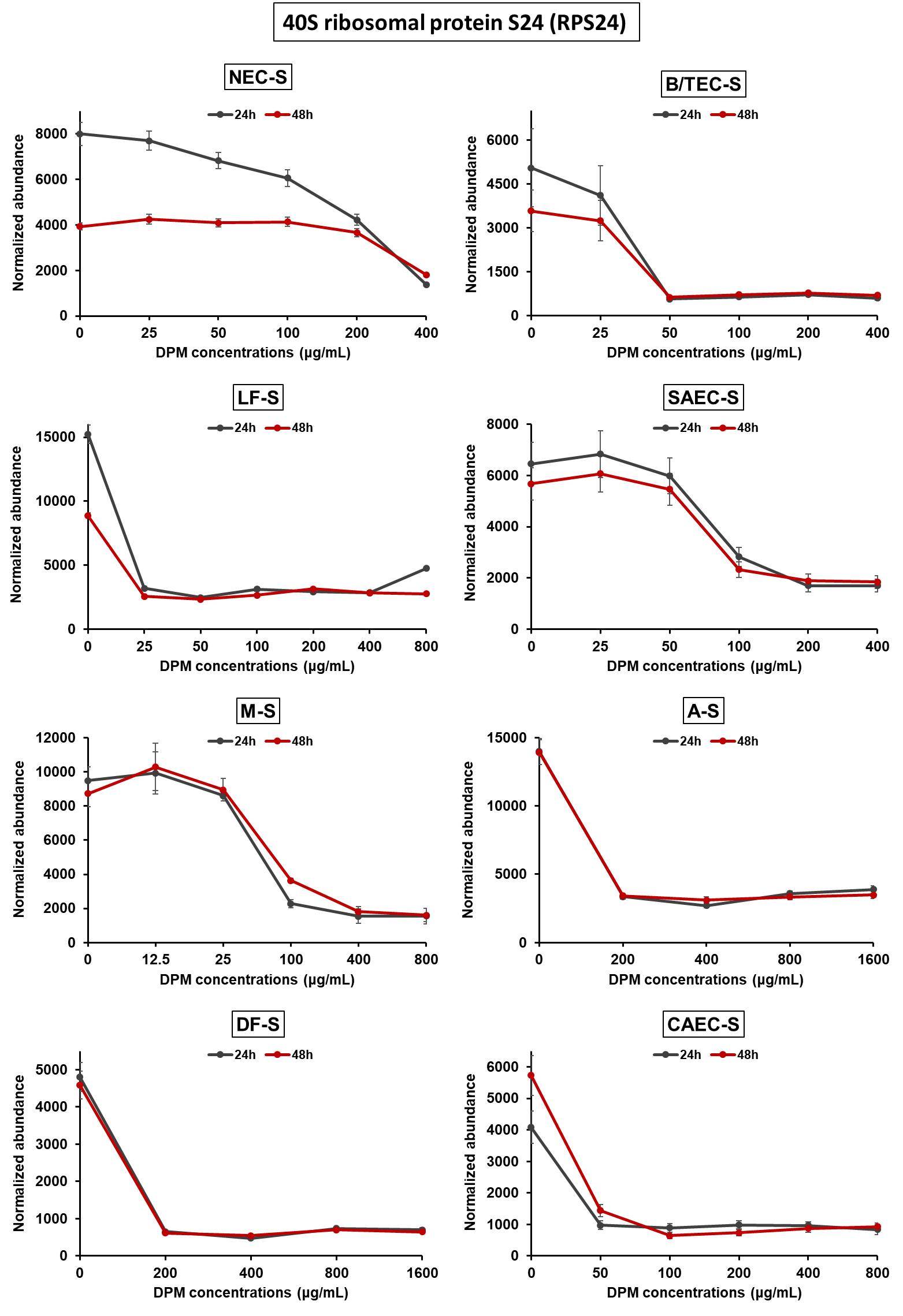


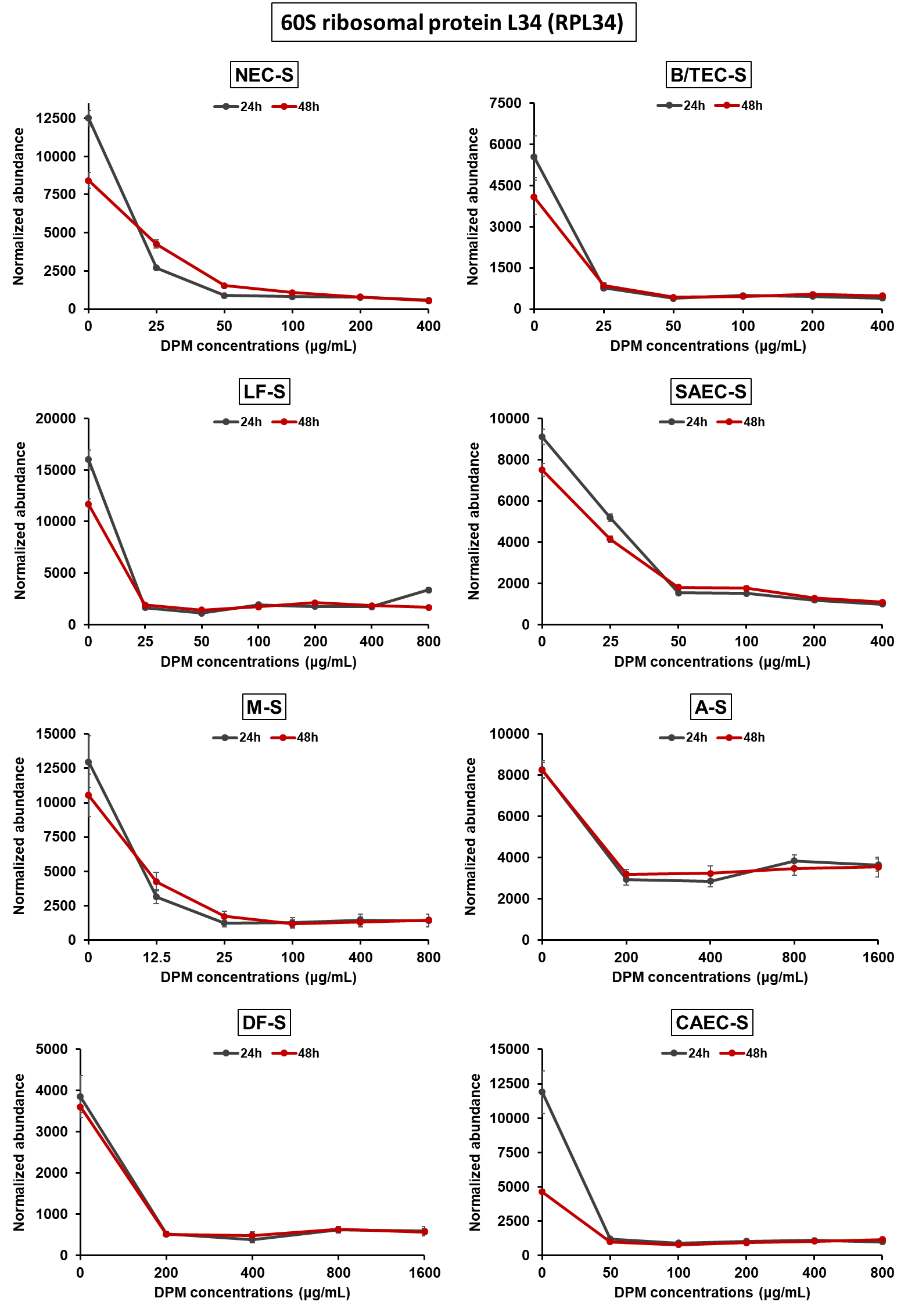


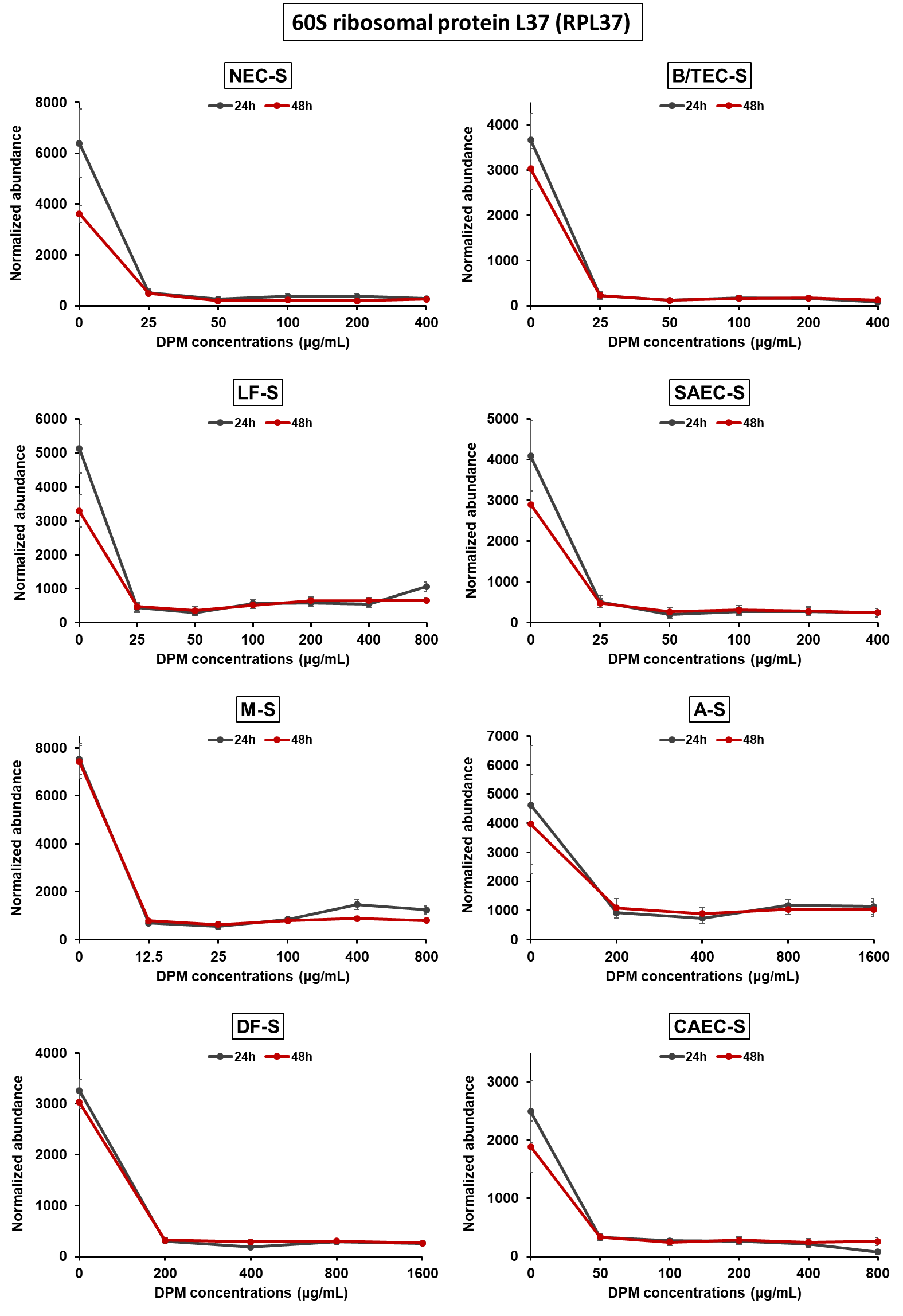


**Supplementary Figure 6**. Abundance levels of seven proteins (beta-2-glycoprotein 1 (APOH), kininogen-1 (KNG1), apolipoprotein C-III (APOC3), and pigment epithelium-derived factor (SERPINF1), 40S ribosomal protein S24 (RPS24), 60S ribosomal protein L34 (RPL34), 60S ribosomal protein L37 (RPL37) in the eight types of spheroids exposed to different DPM concentrations at 24 and 48 h. Data were obtained from three LC-MS/MS runs and are expressed as the mean ± SEM.


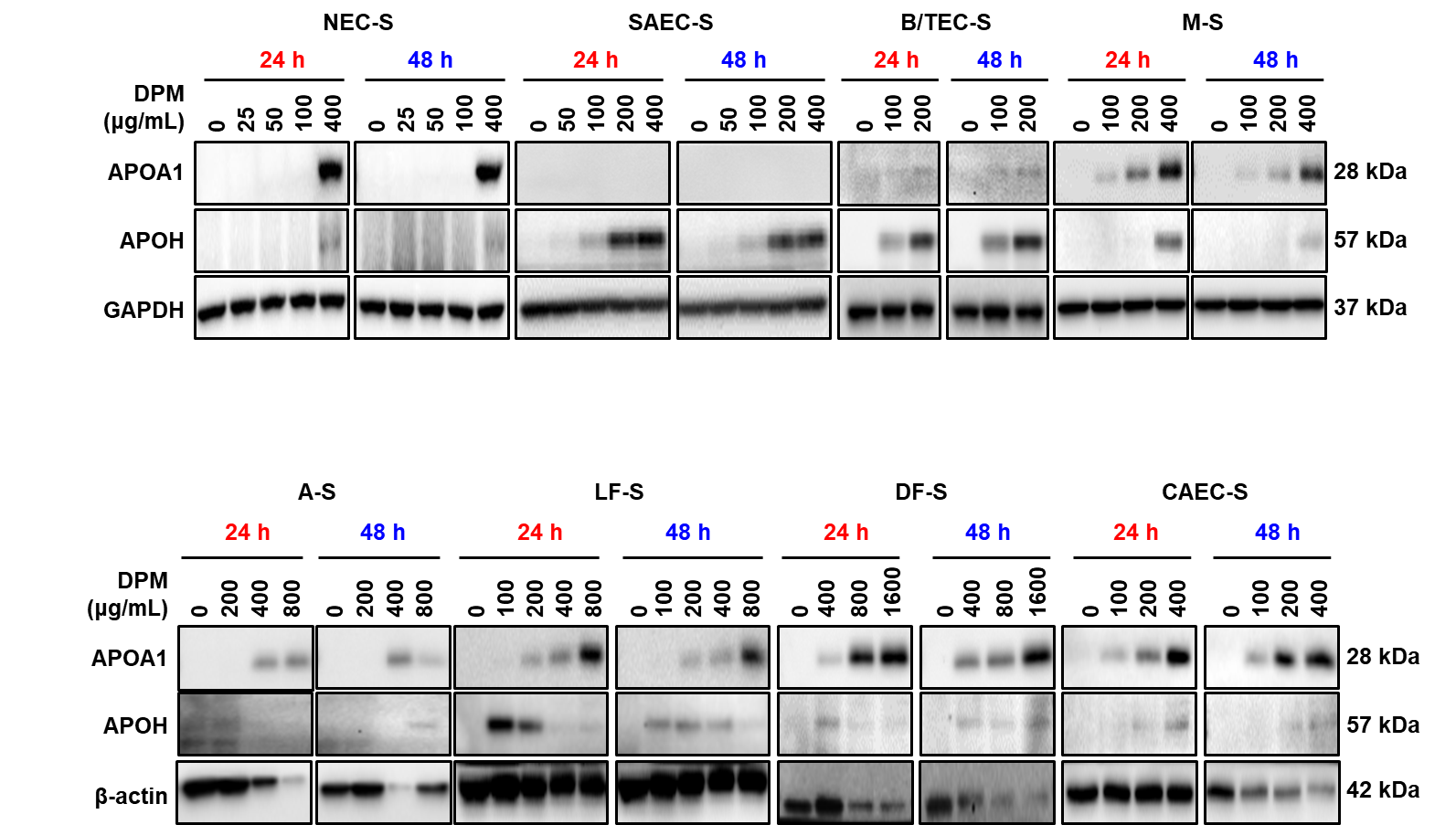


**Supplementary Figure 7.** Western blot analysis of APOA1 and APOH in 8 different spheroids upon DPM treatments**.** 3D spheroids were treated with various concentrations of DPM for 24h or 48 h. The protein expression levels of APOA1 and APOH were determined by western-blot analysis.


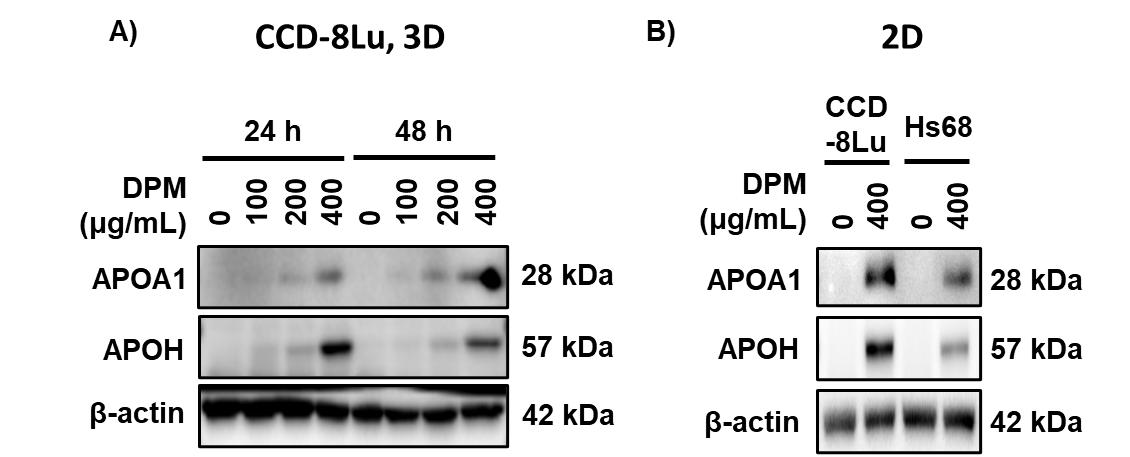


**Supplementary Figure 8.** The effects of DPM on two different fibroblast cell lines, CCD-8Lu and Hs68 cells were similar to those on primary cells cultured in 3D. (A) Western blot analysis of APOA1 and APOH expression in 3D spheroids of CCD-8Lu cells. Cells were incubated with various concentrations of DPM for 24 or 48 h. (B) Western blot analysis of APOA1 and APOH in CCD-8Lu and Hs68 cells cultured on 2D plates. Cells were incubated with or without 400 μg/mL of DPM for 24 h.

**
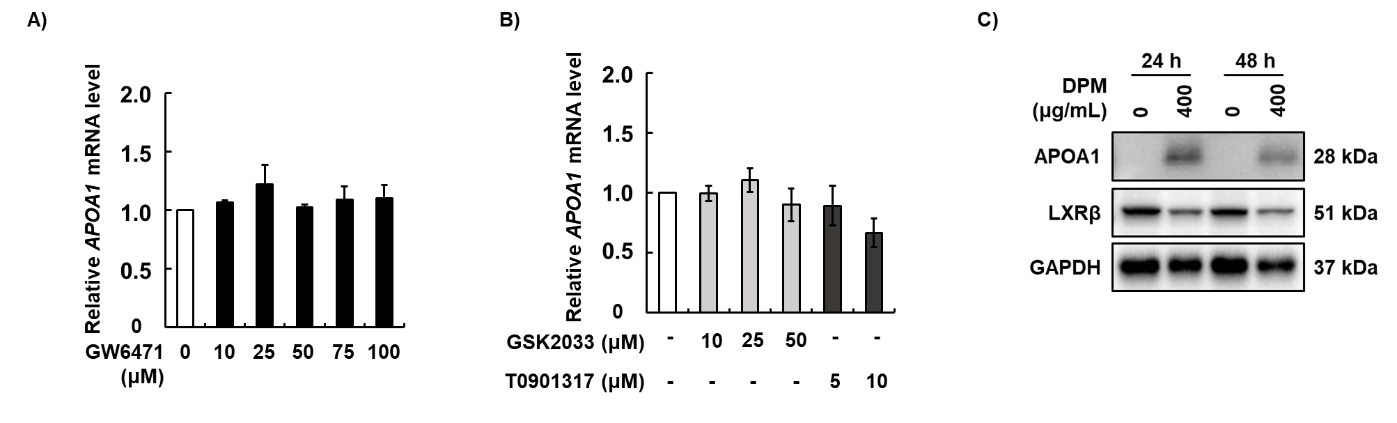
**

**Supplementary Figure 9**. The mRNA of APOA1 in CCD-8Lu cells was not regulated by transcriptional factors, PPARα and LXR. (A) GW6471 (PPARα antagonist), (B) GSK2033 (LXR antagonist) and T0901317 (LXR agonist) were treated for 24 h. RT-qPCR analysis was performed with APOA1 and GAPDH specific primers. (C) Western blot analysis of APOA1, and LXRβ CCD-8Lu cells. CCD-8Lu cells were treated with or without 400 μg/mL DPM for 24 h or 48 h.

**
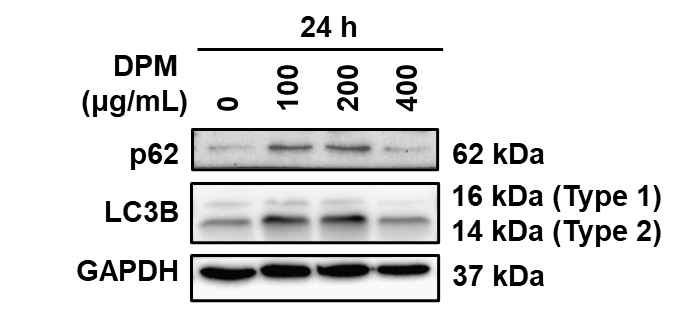
**

**Supplementary Figure 10.**  Protein expression levels of autophagosome complex LC3 and p62 upon DPM treatment in CCD-8Lu cells. CCD-8Lu cells were treated with increasing concentrations of DPM for 24 h.

**Figure 4B**


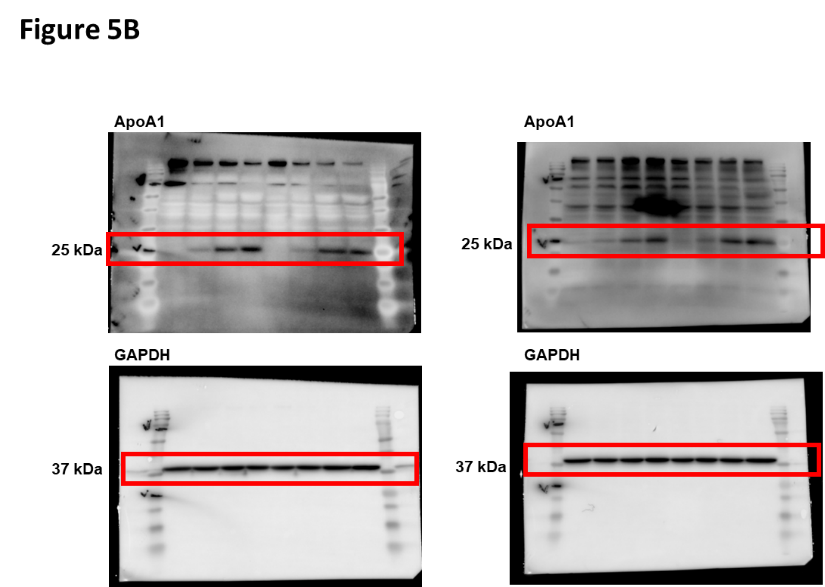


**Figure 5B, 5C**
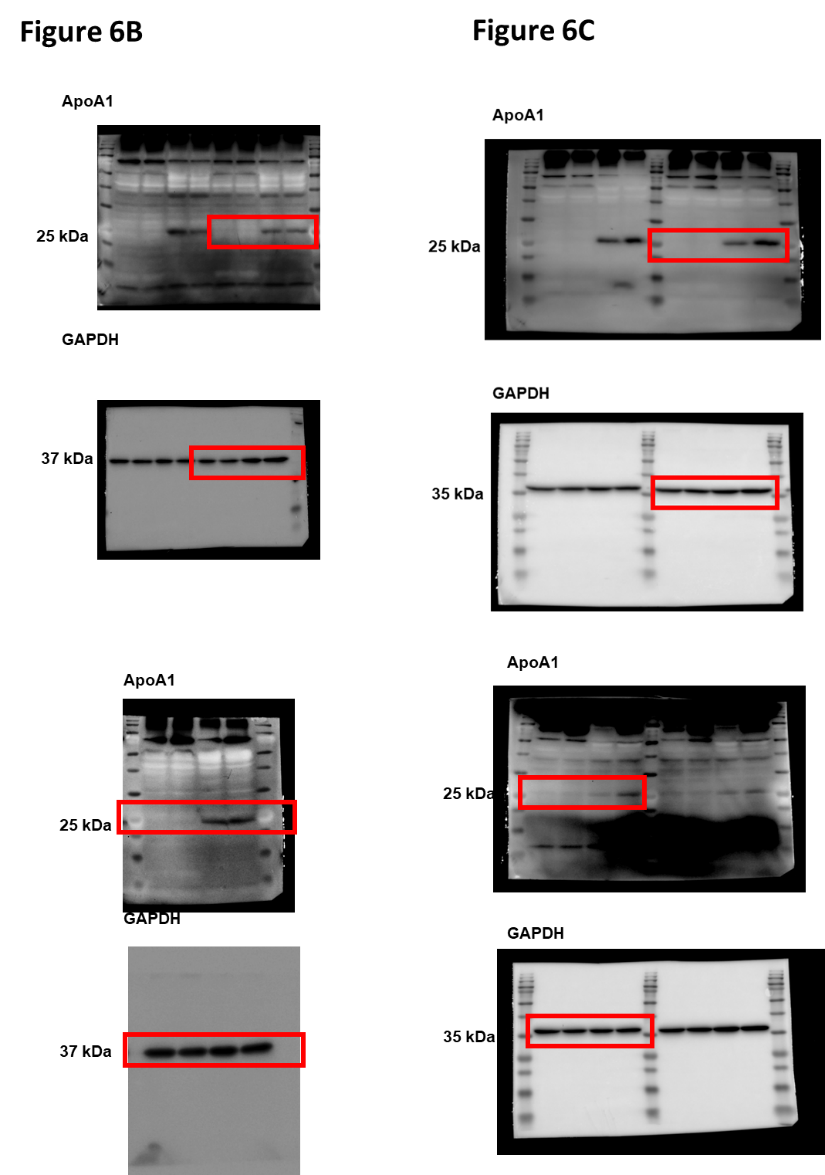


**Figure 6B**


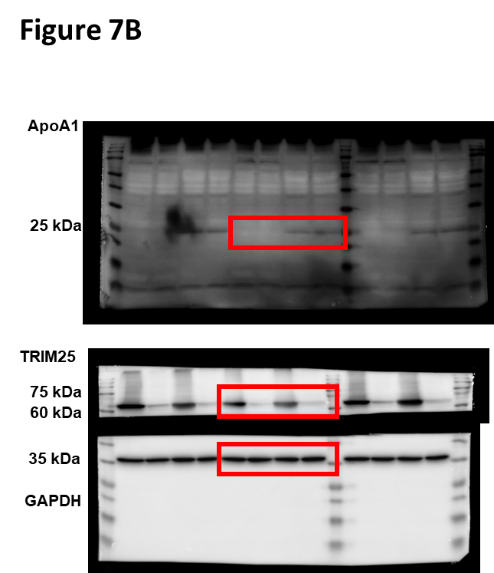


**
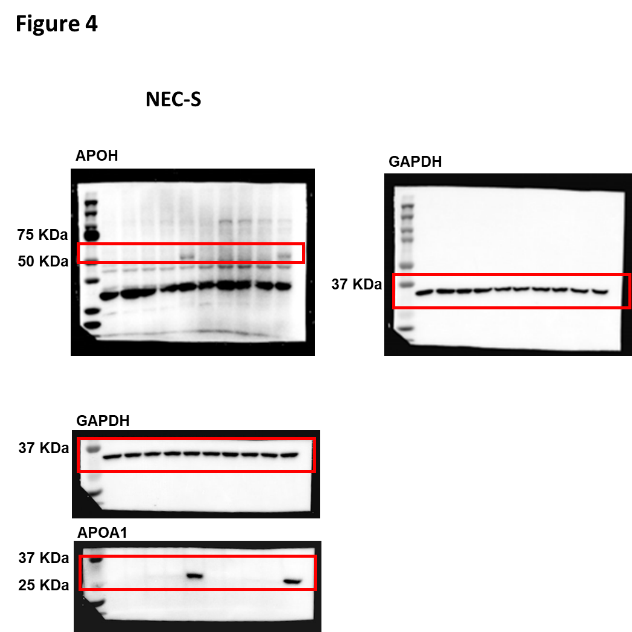

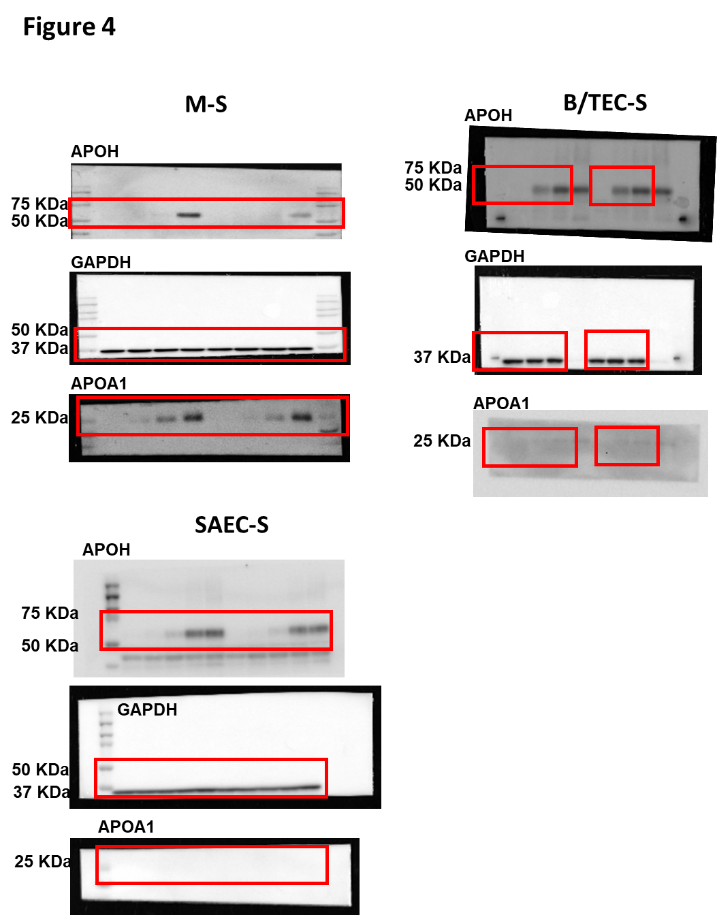
Figure S7**


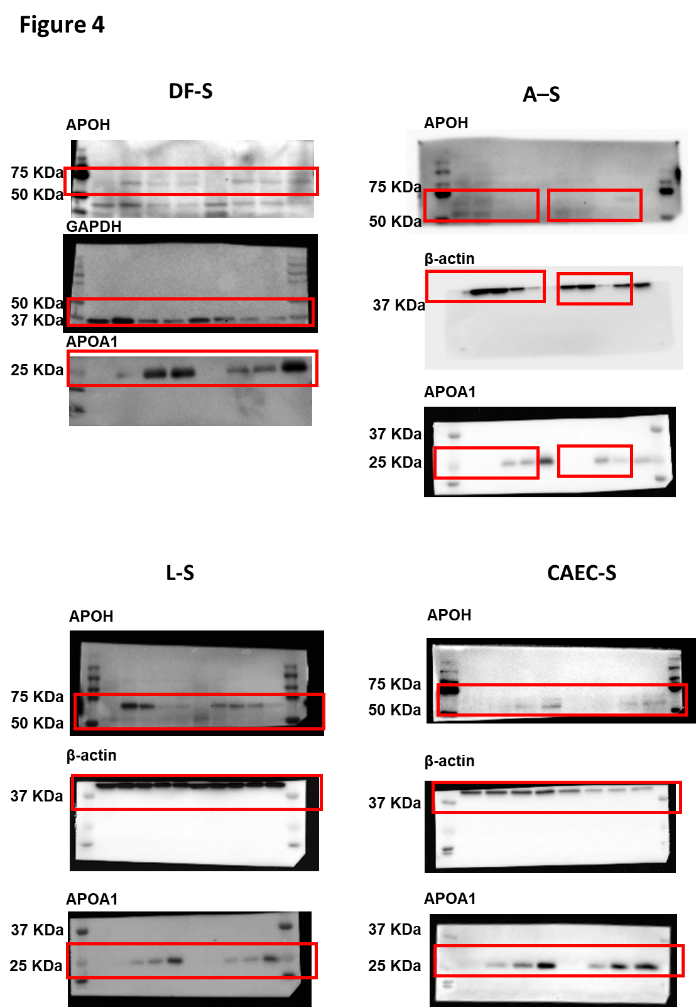


**
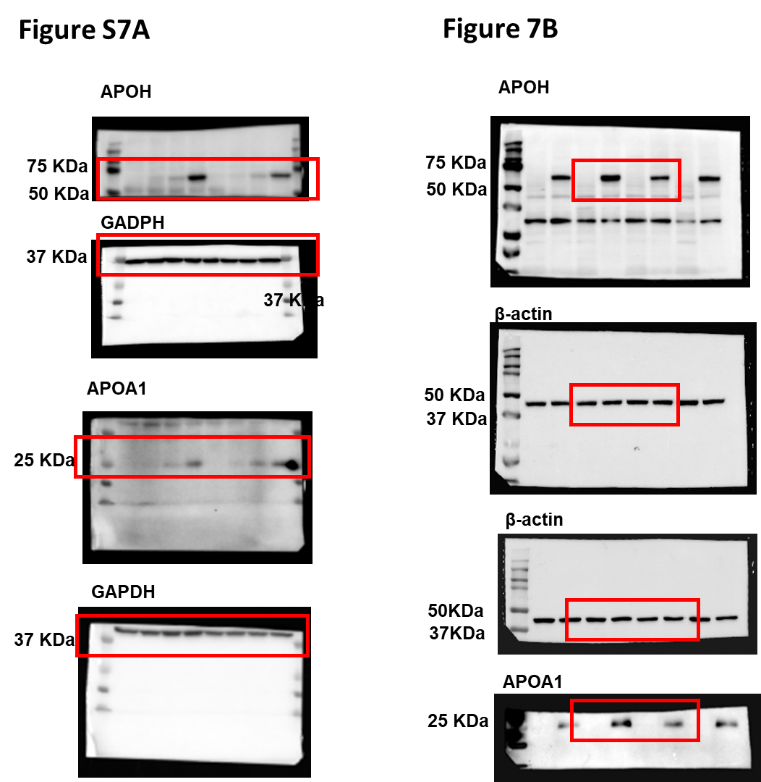
Figure S8**

**Figure S9**


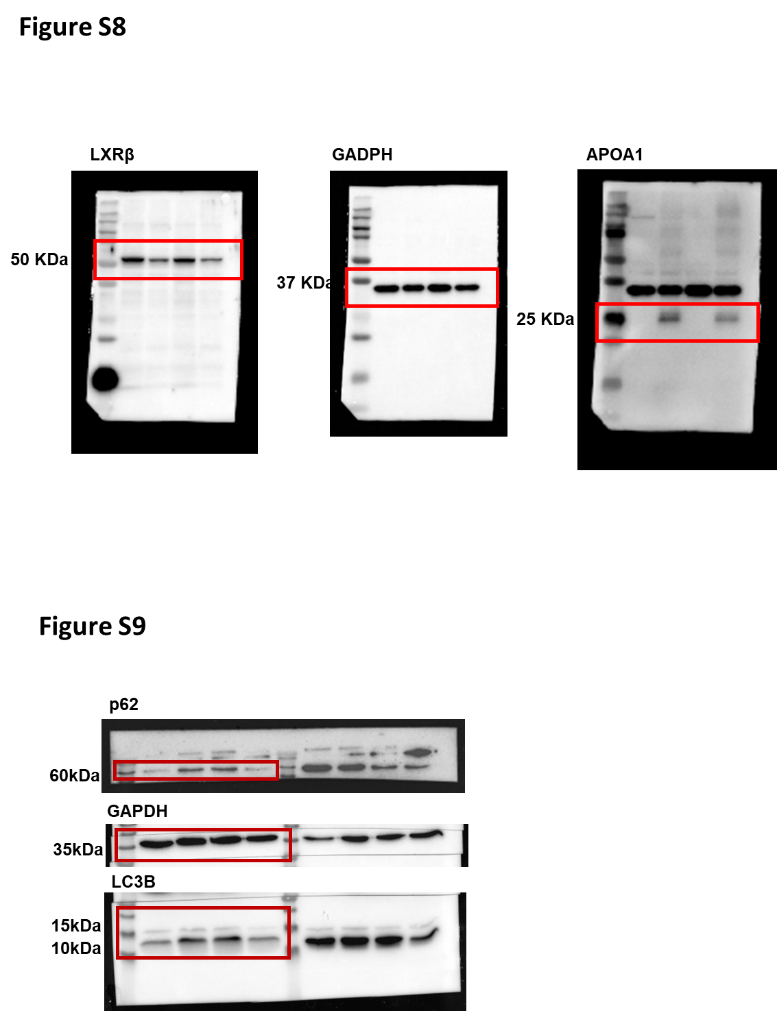


**Figure S10**


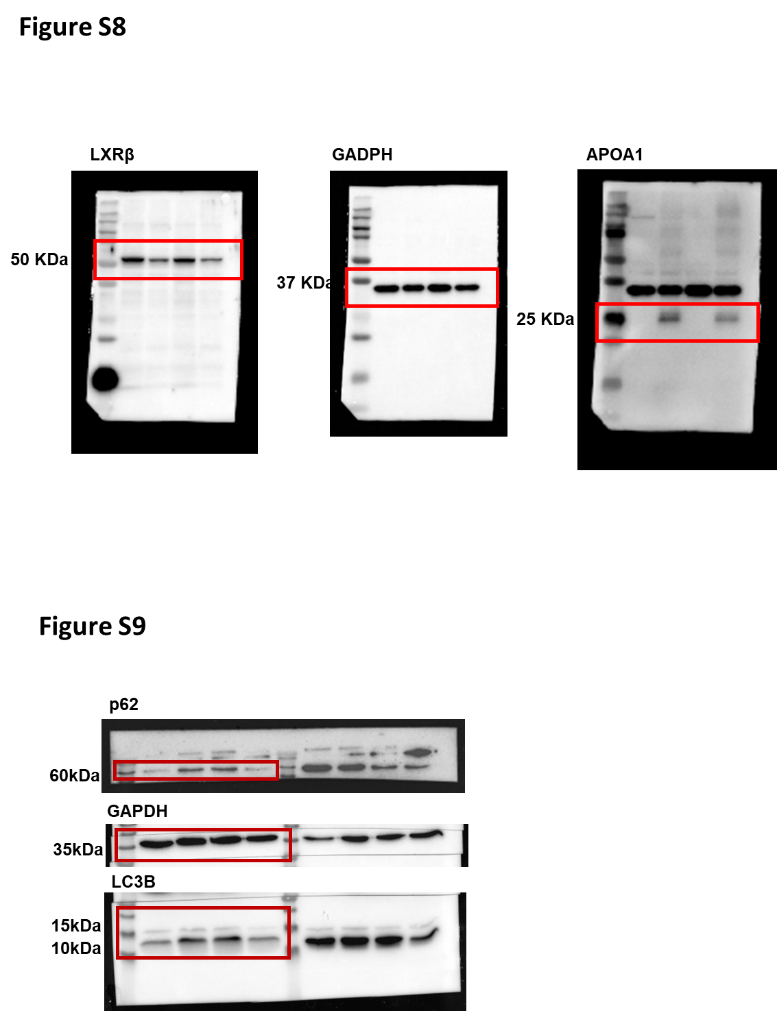


**Supplementary Figure 11.**  The original images of western blot
